# Modulating the mechanism of electrocatalytic CO_2_ reduction by cobalt phthalocyanine through polymer coordination and encapsulation

**DOI:** 10.1038/s41467-019-09626-8

**Published:** 2019-04-11

**Authors:** Yingshuo Liu, Charles C. L. McCrory

**Affiliations:** Department of Chemistry, University of Michigan, Ann Arbor, MI 48109 USA

## Abstract

The selective and efficient electrochemical reduction of CO_2_ to single products is crucial for solar fuels development. Encapsulating molecular catalysts such as cobalt phthalocyanine within coordination polymers such as poly-4-vinylpyridine leads to dramatically increased activity and selectivity for CO_2_ reduction. In this study, we use a combination of kinetic isotope effect and proton inventory studies to explain the observed increase in activity and selectivity upon polymer encapsulation. We provide evidence that axial-coordination from the pyridyl moieties in poly-4-vinylpyridine to the cobalt phthalocyanine complex changes the rate-determining step in the CO_2_ reduction mechanism accounting for the increased activity in the catalyst-polymer composite. Moreover, we show that proton delivery to cobalt centers within the polymer is controlled by a proton relay mechanism that inhibits competitive hydrogen evolution. These mechanistic findings provide design strategies for selective CO_2_ reduction electrocatalysts and serve as a model for understanding the catalytic mechanism of related heterogeneous systems.

## Abstract (web summary)

Understanding the mechanism behind CO_2_ reduction catalysis is crucial in the development of high efficiency and activity catalysts. Here, authors employ kinetic isotope effects and proton inventory studies to assess catalyst mechanism and proton delivery in molecular CO_2_ electroreduction materials.

## Introduction

The selective electrochemical conversion of CO_2_ to value-added products in the CO_2_ reduction reaction (CO_2_RR) offers a promising approach for recycling CO_2_ into value-added products and the storage of intermittent energy sources as chemical fuels^1–4^. State-of-the-art polycrystalline Cu catalysts produce useful products such as methanol, but do so non-selectively and form a variety of other gaseous and liquid products including H_2_ from competitive H^+^ or water reduction^5–8^. Materials such as planar polycrystalline Au^9^ and Ag foils^10^ and some metal-doped nitrogenated carbon materials (MNCs) with M-N_4_ porphyrin-like active sites^11–14^ are more selective for CO_2_ reduction to single C-containing products, primarily CO, but these systems still suffer from the competitive H_2_ evolution reaction (HER). For instance, planar polycrystalline Ag catalysts selectively reduce CO_2_ to CO with >90% Faradaic efficiency at −1.1 V vs RHE, but that selectivity drops to 60% Faradaic efficiency due to competitive H_2_ evolution when the potential changes by 0.2 V in either direction^10^. Similar potential-dependence on product distribution is seen for various MNC materials^12,14,15^. Therefore, the discovery of systems that preferentially promote selective CO_2_ reduction to single products with high activity while suppressing HER is critically important for the realization of selective electrochemical CO_2_ reduction.

Our research approach is to encapsulate molecular catalysts within coordinating polymers to promote selective CO_2_ reduction. By encapsulating the molecular catalysts within the coordinating polymers, we are able to not only control H^+^ and CO_2_ delivery to the catalyst centers, but also tune catalytic activity through primary-, secondary-, and outer-coordination sphere effects. These polymer-catalyst composite systems are inspired by enzymatic systems such as NiFe carbon monoxide dehydrogenase and FeFe hydrogenase where fast catalytic activity and high product selectivity are achieved by carefully controlling the primary-, secondary-, and outer-coordination spheres of the enzyme’s active site^16–18^.

**Fig. 1:**
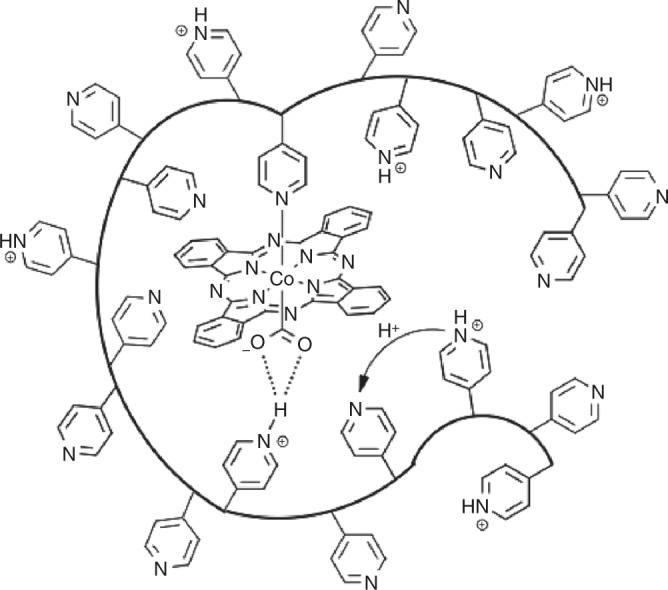
An illustration of a cobalt phthalocyanine (CoPc) encapsulated within a hydrophobic poly-4-vinylpyridine (P4VP) membrane highlighting the postulated primary-, secondary-, and outer-coordination sphere effects^19^

Our initial studies focused on encapsulating cobalt phthalocyanine (CoPc) within the coordinating polymer poly-4-vinylpyridine (P4VP)^19^. When adsorbed onto graphite electrodes without a polymer binder, CoPc by itself is a non-selective CO_2_RR catalyst that shows only modest activity for CO_2_ reduction to CO in aqueous citrate and phosphate solutions accompanied by significant co-generation of H_2_ from the competitive HER^19–22^. Previous studies have also shown that incorporation of CoPc within P4VP adsorbed onto graphite electrodes results in increased activity and selectivity for CO_2_ reduction over competitive HER compared to the parent CoPc complex^19,20,22^. It has been postulated that the enhanced activity and selectivity is due to three synergistic effects: (1) axial-coordination of pyridyl to the Co center in the primary-coordination sphere increasing the catalyst’s nucleophilicity for CO_2_ binding, (2) H-bonding interactions in the secondary-coordination sphere that stabilize reactive CO_2_ intermediates, and (3) control of proton delivery through the use of the pyridyl residues within the polymer as proton relays in the outer-coordination sphere (Fig. 1)^19,20^. We propose that axial coordination of pyridyl facilitates CO_2_ coordination and thus changes the rate-determining step of CO_2_RR by five-coordinate CoPc-P4VP systems to a step subsequent to CO_2_ coordination. In addition, the increase in selectivity for CO_2_RR over HER could be due to a weak acid effect from the protonated pyridyl residues on the proton relays. The protonated pyridyl residues in the polymer may be acidic enough to act as a proton relay and donor to the activated CO_2_ intermediate, but proton transport through the polymer may be sluggish enough to suppress HER activity. Other recent studies have supported the assertion that synergy between catalyst and polymer effects is required for increased activity and selectivity in polymer-encapsulated systems^23–31^.

In this work, we expand upon our previous studies of the CoPc-polymer systems to explicitly investigate the mechanistic implications of primary- and outer-coordination sphere effects on the CO_2_ reduction activity by catalyst-polymer composites. To do this, we use a combination of kinetic isotope effect (KIE) measurements and proton inventory studies to determine both the involvement of protons in the rate-determining step of the catalytic mechanism and the mechanism of H^+^ transport through the polymer chain as we systematically alter the nature of the CoPc-polymer interactions. We observe a difference in the measured KIE for the four-coordinate CoPc systems (such as CoPc and CoPc-P2VP) compared to the five-coordinate systems (such as CoPc(py) and CoPc-P4VP) that is consistent with a change in the rate-determining step of the mechanism from CO_2_ binding step to a subsequent protonation of the coordinated CO_2_ intermediate. In addition, using proton inventory studies—a technique that is used in enzymology to study the kinetics of proton delivery to enzymatic active centers based on the attenuation of kinetic rates as a function of fractional solvent deuteration^32–35^—we show that proton-transport to the Co active site in CoPc-P4VP and related systems is controlled by proton relays in the polymer rather than diffusion through the film. Thus, we provide direct experimental evidence that proton relays in the outer-coordination sphere of the catalyst in CoPc-P4VP play an important role in promoting selective catalytic activity as has been suggested for other synthetic molecular and enzymatic systems^36,37^. We believe this work is among the first examples of extending proton inventory studies from traditional enzymological systems to electrocatalytic studies in synthetic molecular-based assemblies^38^. Our studies help us to better understand the CO_2_ reduction mechanism of polymer-encapsulated catalysts for comparison to related MNC and planar metal catalyst systems and more generally provides a strategy to probe fundamental catalytic mechanism of CO_2_ reduction by molecular assemblies using KIE and proton inventory measurements.

## Results

### Surface immobilized catalysts and catalyst-polymer systems

**Fig. 2:**
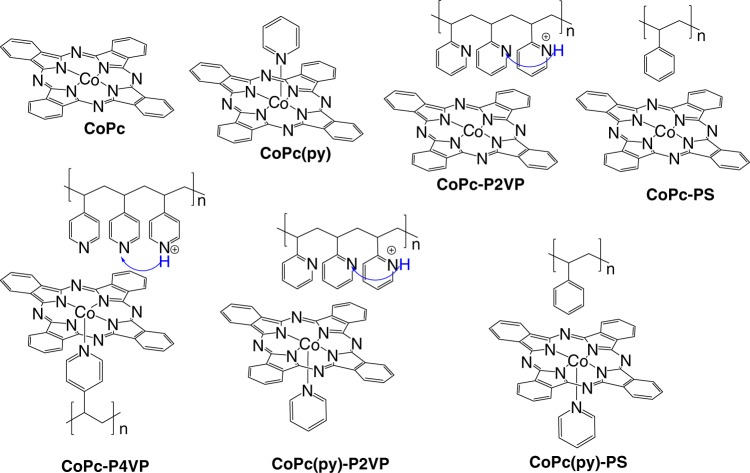
Catalyst and polymer-catalyst composite systems investigated in this work along with their postulated coordination environment and proton relays

To determine both the involvement of protons in the rate-determining step of the catalytic mechanism and the mechanism of H^+^ transport through the polymer chain, we use a combination of KIE measurements and proton inventory studies on different catalytic systems as we systematically alter the nature of the CoPc-polymer interactions (Fig. 2). All catalysts and catalyst-polymer composite systems studied were surface-immobilized by drop-casting a catalyst film directly onto edge-plane graphite (EPG) disk electrodes and drying at 70 °C as described in the Supplementary Methods section in the Supplementary Information. For each system, plots of peak area as a function of scan rate for the non-catalytic [CoPc]^+^/[CoPc] peak are linear (Supplementary Figs. 1–14) which is consistent with a surface-immobilized species. The Co loading of each system was calculated to be 2.19 × 10^−9^ mol cm^−2^ based on the deposition procedures and this was confirmed by dissolving the catalyst film from the surface into 1 M HNO_3_ aqueous solution and then measuring the concentration in the resulting solution with ICP-MS (Supplementary Table 1).

### Proposed CO_2_RR mechanisms by CoPc

**Fig. 3:**
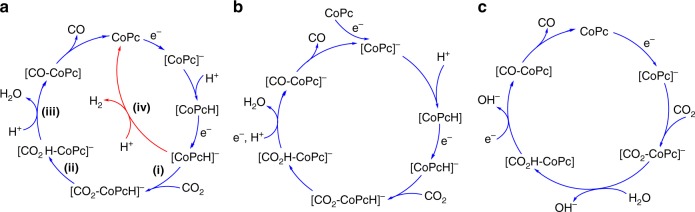
Proposed CO_2_ reduction mechanisms of CoPc in this work and other proposed mechanisms. **a** A proposed mechanism for CO_2_ reduction by CoPc showing pathway for competitive H_2_ generation^20–22^. Note that we do not assign individual oxidation states to the Co center and instead refer to the overall charge on the entire complex. Reported molecular orbital calculations of CoPc suggest that the first reduction may be a metal-centered reduction of Co^II^Pc to Co^I^Pc followed by a second ligand-based reduction^41^. Other proposed CO_2_ reduction by CoPc in **b**, organic solutions^20^ and **c**, low concentration bicarbonate buffer in aqueous solution^39,40^

The exact mechanism for electrocatalytic CO_2_ reduction by CoPc remains a point of discussion within the community. Based on previously reported experimental evidence, a proposed mechanism for CO_2_ reduction by CoPc with the competitive HER pathway is shown in Fig. 3a^20,22^. In the proposed mechanism, CoPc is first reduced to [CoPc]^−^ followed by protonation of the complex (presumably on the Pc ring) to form [CoPcH] and a second reduction to produce [CoPcH]^−^. Here, there is a branch in the mechanism where [CoPcH]^−^ can either react with H^+^ to evolve H_2_ and regenerate the CoPc starting material in step (iv), or [CoPcH]^−^ can react with CO_2_ to form a CO_2_ adduct in step (i) that, upon subsequent protonation in step (iii), generates CO^20,22^. This mechanism is consistent with previous results for CoPc and CoPc-P4VP in phosphate solutions which show the onset of catalytic activity occurs at the second reduction event in the voltammogram^19,22^. However, our recent electrochemical study of CoPc in DMSO solutions suggest that under conditions of low H^+^ activity, a third reduction event is required for catalytic turnover of CO_2_RR (see Supplementary Fig. 15). This is consistent with a previous spectroelectrochemical studies in organic systems under CO_2_, which suggests that further reduction of the [CoPc-CO] adduct is required to release CO and re-enter the catalytic cycle at [CoPc]^−^ (Fig. 3b)^20^. Alternatively, recent reports of CO_2_ reduction by adsorbed CoPc in bicarbonate solutions have suggested that CO_2_ coordination may occur at the 1 e− reduced species^39^, and this pathway has been further supported by a recent Tafel analysis and DFT studies (Fig. 3c)^40^. Although we cannot distinguish between the three mechanisms, all support our postulate that promotion of CO_2_ reduction over competitive H_2_ evolution can be achieved by either (a) facilitating CO_2_-coordination or (b) controlling H^+^ delivery to the active site to inhibit the competitive H_2_ evolution pathway. In addition, all three pathways are consistent with our KIE and proton inventory studies discussed below. The mechanistic discussions in the manuscript will focus on the mechanism shown in Fig. 3a because it is the mechanism that has been proposed to operate under our reaction conditions.

### KIE studies

KIE studies were conducted to investigate the influence of axial ligand coordination to CoPc on the CO_2_RR mechanism. The magnitude of KIE is given by Eq. (1), where *j*_H_ is the electrocatalytic current density measured in the protic solution and *j*_D_ is the electrocatalytic current density measured in the deuterated solution:

1 $${\mathrm{KIE}} = \frac{{j_{\mathrm{H}}}}{{j_{\mathrm{D}}}}$$

**Fig. 4:**
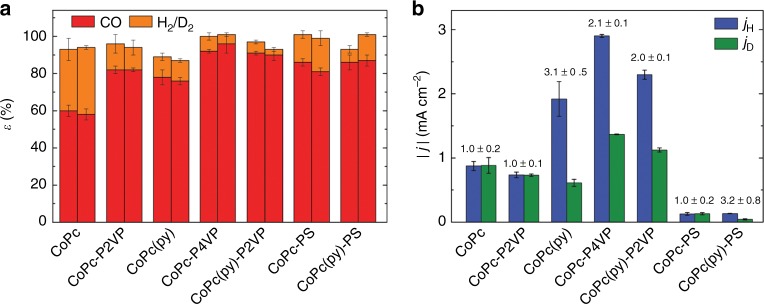
CO_2_ reduction performance in protic and deuterated solutions. **a** Faradaic efficiencies (*ɛ*) of 2-h controlled potential electrolyses at −1.25 V vs. SCE for H_2_/D_2_ (orange) and CO (red) in protic electrolyte (left bar), and in deuterated electrolyte (right bar). **b** Measured current densities at −1.25 V vs. SCE in protic electrolyte (blue bar) and deuterated electrolyte (green bar) for each of the systems shown in Fig. 2. Kinetic isotope effect values are listed above the bars and also summarized in Table 1. All reported values are averages from three or more independent measurements, and all errors are given as standard deviations.

Note that Eq. (1) assumes that the electrochemical reaction rate is directly proportional to the measured current density which is generally expected for a reaction occurring at a surface-immobilized species^42–44^. However, Eq. (1) is valid for determining KIE only for systems in which the Faradaic efficiency, ε, is the same for an electrocatalytic reaction conducted in protic and deuterated solvent. To confirm that the Faradaic efficiency for CO production does not change as the electrolyte is changed from a protic solution to a deuterated solution, we conducted 2-h controlled potential electrolyses (CPE) measurements in sealed electrochemical cell and measured the Faradaic efficiencies for CO and H_2_/D_2_ (Fig. 4a, Supplementary Table 2) for each system. In general, the Faradaic efficiencies for a given system do not change as we change from a protic electrolyte to a deuterated electrolyte, validating our use of Eq. (1) for determining the KIE. Note that longer-term 8-h CPE measurements show equivalent Faradaic efficiency and minimal loss of activity suggesting the catalyst systems investigated in this study are relatively stable under the reaction conditions (see Supplementary Table 3 and Supplementary Fig. 16).

**Table 1:** Activity and Faradaic efficiency (ε) measurements for catalysts in protic and deuterated solutions, and determined kinetic isotope effects

Catalyst	*j*_H, D_ (mA∙cm^−2^)	TOF_CO H,D_^d^ (s^−1^)	ε_CO,H_ (%)	ε_CO,D_ (%)	KIE	Proton inventory parameters	
						*ϕ*	*Z*
CoPc	(H)^b^ −0.87 ± 0.07	(H) 1.24 ± 0.12	60 ± 3	58 ± 3	1.0 ± 0.2	─^e^	─^e^
	(D)^c^−0.88 ± 0.12	(D) 1.21 ± 0.18					
CoPc-P2VP^a^	(H) −0.73 ± 0.04	(H) 1.42 ± 0.09	82 ± 2	82 ± 1	1.0 ± 0.1	─^e^	─^e^
	(D) −0.73 ± 0.02	(D) 1.41 ± 0.04					
CoPc(py)	(H) −1.92 ± 0.27	(H) 3.54 ± 0.53	78 ± 4	76 ± 2	3.1 ± 0.5	0.30 ± 0.01	1.02 ± 0.02
	(D) −0.61 ± 0.06	(D) 1.10 ± 0.11					
CoPc-P4VP^a^	(H) −2.90 ± 0.02	(H) 6.31 ± 0.08	92 ± 1	96 ± 5	2.1 ± 0.1	0.29 ± 0.01	1.65 ± 0.03
	(D) −1.37 ± 0.01	(D) 3.10 ± 0.16					
CoPc(py)-P2VP^a^	(H) −2.30 ± 0.07	(H) 4.94 ± 0.16	91 ± 1	90 ± 3	2.0 ± 0.1	0.30 ± 0.01	1.60 ± 0.03
	(D) −1.12 ± 0.03	(D) 2.39 ± 0.10					
CoPc-PS^a^	(H)−0.13 ± 0.02	(H) 0.26 ± 0.04	86 ± 2	81 ± 2	1.0 ± 0.2	─^e^	─^e^
	(D) −0.13 ± 0.02	(D) 0.25 ± 0.03					
CoPc(py)-PS^a^	(H) −0.13 ± 0.00	(H) 0.27 ± 0.01	86 ± 4	87 ± 3	3.2 ± 0.8	0.29 ± 0.02	1.06 ± 0.04
	(D) −0.04 ± 0.01	(D) 0.09 ± 0.02					

All measurements conducted at −1.25 V vs. SCE. All reported values are averages from three or more independent measurements, and all errors are given as standard deviations

^a^Polymer-catalyst composite films were drop-cast from deposition solutions containing 1 % w/v polymer

^b^Under protic condition

^c^Under deuterated condition

^d^Turnover frequencies for CO (TOF_CO_) is calculated from both the overall activity measured in rotating disk chronoamperometric (CA) steps at −1.25 V vs. SCE and the faradaic efficiencies measured in 2-h controlled potential electrolyses (CPE) (see Supplementary Information for detailed explanation)

^e^Not measured

In general, a KIE > 1 suggests that a proton transfer event is present in the rate-determining step of the mechanism, whereas a KIE ≈ 1 suggests no involvement of a formal proton-transfer event in the rate-determining step^32,45–47^. Thus, evaluation of the KIE for the adsorbed CoPc parent complex and our modified CoPc systems can provide information regarding the rate-determining step in the proposed electrocatalytic CO_2_RR mechanism shown in Fig. 3a. To determine the KIE for our systems, we measured the electrocatalytic current for CO_2_RR both in pH 5 phosphate solution and in pD 5 deuterated phosphate solution using 2-min rotating disk chronoamperometric (CA) step measurements at −1.25 V vs. SCE and a rotation rate of 1600 rpm (representative CA measurements for each system investigated are shown in Supplementary Figs. 17–27). The measured current densities and KIE values are reported in Fig. 4b, and all the KIE study results for CO_2_RR are summarized in Table 1. Note that ICP-MS measurements show no difference in Co loading on samples measured pre-CA measurements and identically-prepared samples measured post-CA measurements (Supplementary Table 1). This suggests there is no loss of Co during the electrolyses.

The parent four-coordinate CoPc system shows no KIE in our studies which is consistent with a rate-determining CO_2_-coordination step (Fig. 3a, step (i)). In the case of CoPc-P2VP, the CoPc complex is immobilized within a non-coordinating P2VP polymer and again, there is no observed KIE. In contrast, the five-coordinate CoPc(py) system shows a KIE = 3.1, suggesting a rate-determining proton transfer step in the mechanism (Fig. 3a, step (iii)). When the five-coordinate CoPc(py) is immobilized within P2VP to form CoPc(py)-P2VP, we observe a smaller KIE = 2.0 compared to the CoPc(py). Likewise, when CoPc is immobilized within the coordinating polymer P4VP to form CoPc-P4VP, the observed KIE = 2.1. The results with CoPc-P4VP and CoPc(py)-P2VP suggest that the polyvinylpyridine polymers are moderating the extent of KIE for the five-coordinate CoPc systems with axially-ligated pyridyls. To confirm that this moderation of the KIE is specifically due to the polyvinylpyridine and not a general effect with any polymer, we measured the KIE of CoPc and CoPc(py) immobilized within polystyrene (PS) where we expect no primary-, secondary-, or outer-coordination sphere effects within the polymer. In this case, CoPc-PS shows no KIE, and CoPc(py)-PS has KIE = 3.2, which is nearly identical to that of CoPc(py) without an encapsulating polymer. Based on the larger KIE for CoPc(py) compared to that of CoPc-P4VP and CoPc(py)-P2VP, we hypothesize that the pyridyls in the polymer act as a proton relay controlling proton delivery to the CoPc active sites, and these sites have a weak inverse isotope effect (KIE < 1) that, in aggregate, moderates the overall KIE for CoPc-P4VP and CoPc(py)-P2VP compared to CoPc(py). Note that additional KIE measurements for HER for the catalysts studied in this work were also measured, and these results are summarized in Supplementary Table 4.

To confirm the five-coordinate nature of the CoPc in CoPc(py), CoPc-P4VP, CoPc(py)-P2VP, CoPc(py)-PS, and CoPc(py)-P2VP, we conducted UV–Vis spectroscopy studies of drop-cast films to characterize the coordination environment (see Supplementary Information for more details). As shown in Supplementary Fig. 28, the Q band of CoPc in PS and P2VP near 669 nm is red shifted to 674 nm in UV–Vis absorption spectrum of CoPc in P4VP, and CoPc(py) in PS and P2VP films. Similarly, red shifted Q bands are also exhibited in the UV–Vis spectra of solutions of CoPc(py) as prepared for deposition and solutions of independently synthesized CoPc(py) by about 5 nm compared to that of CoPc (Supplementary Fig. 29). These red shifts are consistent with the observation that the Q bands of metalloporphyrin-like complexes red shift when there are electron-donating ligands coordinated axially with the central metal ions^48,49,50^. Thus, the UV–Vis spectrum suggests that CoPc exists as a five-coordinate complex in CoPc-P4VP, CoPc(py)-PS, CoPc(py)-P2VP samples, and CoPc(py) solutions, which is consistent with axial coordination of pyridyl groups to the CoPc in these systems.

Recent studies have suggested that CoPc aggregation occurs when CoPc is adsorbed to carbon surfaces at high loadings^39,51,52^ and that this aggregation limits the number of exposed active sites and, therefore, the measured per-CoPc TOFs^39^. To explore whether aggregation influences the results of our mechanistic studies, we explored the loading dependence on CO_2_RR activity by CoPc both physisorbed onto EPG and within the P4VP films over four orders of magnitude of loading between 2.19 × 10^−11^ mol cm^−2^ and 2.19 × 10^−7^ mol cm^−2^ (results are summarized in Supplementary Figs. 30–32). We observe a decrease in TOF for CO_2_RR with increasing CoPc loading consistent with previous aggregation studies^39^. However, importantly the KIE results are statistically equivalent at every loading suggesting that aggregation does not change the rate-determining step in the catalytic mechanism (see Supplementary Table 5).

To confirm that the observed increased activity for CoPc(py) and CoPc-P4VP compared to the parent CoPc system is not due to electrocatalytic CO_2_ reduction by free pyridine in solution or the polymer pyridyl groups, we conducted several control experiments. We have previously conducted CPE experiments with CoPc-modified carbon electrodes in CO_2_-saturated pH 5 phosphate solutions containing 0.05 mM dissolved pyridine and saw no significant change in the CO_2_RR activity and Faradaic efficiency compared to analogous studies with no dissolved pyridine present^19^. In addition, we have previously shown that CPE experiments conducted with EPG electrodes coated with P4VP (with no CoPc) showed negligible CO_2_RR activity^19^. In this work we have conducted additional CPE experiments with bare EPG electrodes in CO_2_-saturated pH 5 phosphate solutions containing 0.05 mM dissolved pyridine and see negligible activity for CO_2_RR (Supplementary Table 6). Based on the results of these control experiments, we conclude that the enhanced current we observe in the CoPc(py) and CoPc-P4VP systems is not due to direct electrocatalytic CO_2_ reduction by free pyridine and/or the pyridyl moieties in the P4VP polymer.

### Proton inventory studies

Although we invoke the existence of proton relays to help explain trends in catalyst selectivity and activity in the CoPc-P4VP and related systems, traditional KIE measurements are not sufficient to definitively argue for their existence. To provide further support for the existence of proton relays within our catalyst-polymer systems we have conducted electrochemical proton inventory studies. The proton inventory method is a technique that is used in enzymology to study the kinetics of proton delivery to enzymatic active centers in which the attenuation of a kinetic rate is measured as a function of the fractional concentration of D_2_O in a mixed D_2_O–H_2_O solvent^32–35^. The method is particularly useful for resolving the number of exchangeable hydrogenic sites that contribute to the catalytic rate within a system^53–55^. The dependence of the rate attenuation on fractional deuteration of the electrolyte can be expressed with a modified Gross-Butler equation (Eq. (2)).

2 $$j_n = j_0(1 - n + n\phi )Z^n$$

3 $$n = \frac{{[{\mathrm{D}}_2{\mathrm{O}}]}}{{\left[ {{\mathrm{D}}_2{\mathrm{O}}} \right] + [{\mathrm{H}}_2{\mathrm{O}}]}}$$

Here *j*_*n*_ is the measured current density at a given fractional deuteration concentration *n* (Eq. (3)), *j*_0_ is the measured current density in solutions with only protic electrolyte present, *ϕ* is the isotopic fractionation parameter which is related to the propensity for a hydrogenic site in the rate-determining step of the reaction to interact with D^+^ compared to water, and *Z* is a parameter related to the aggregate isotope effect from multiple equivalent hydrogenic sites, called *Z*-sites, with individual weak isotope effects (see Supplementary Information for an explanation of Eq. (2))^33,44,53^.

**Fig. 5:**
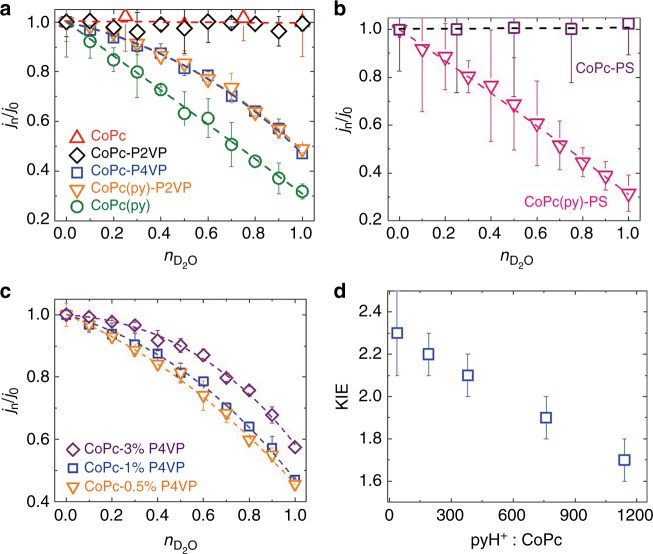
Proton Inventory Studies of CoPc and Related systems. **a** Proton inventory studies of CO_2_ reduction by CoPc (red triangles), CoPc-P2VP (black diamonds), CoPc-P4VP (blue squares), CoPc(py)-P2VP (orange triangles) and CoPc(py) (green circles). The red dashed line is the guide to the eye for CoPc and CoPc-P2VP (*j*_n_ = *j*_0_ at every n measured), and the blue, orange and green dashed lines are fits to the data using equation (2). The resulting values for *ϕ* and *Z* are shown in Table 1. Note that KIE = *j*_H_/*j*_D_. **b** Proton inventory studies of CO_2_ reduction by CoPc-PS (purple squares) and CoPc(py)-PS (pink triangles). The purple dashed line is the guide to the eye for CoPc-PS case (*j*_n_ = *j*_0_ at every *n* measured), and the pink dashed line is a fit to the data using Eq. (2). The resulting values for *ϕ* and *Z* are shown in Table 1 Note that KIE = *j*_H_/*j*_D_. **c** Proton inventory studies of CO_2_ reduction by CoPc-P4VP drop-cast from deposition solutions with different P4VP loadings: 0.5 % w/v (orange triangles), 1% w/v (blue squares), and 3% w/v (purple diamonds). The dashed lines are fits to the data by Eq. (2) and the resulting values for *ϕ* and *Z* are shown in Table 2. **d** KIE value decreases with increasing the pyH^+^: CoPc ratio in the polymer. All reported values are averages from three or more independent measurements, and all errors are given as standard deviations.

For all the systems investigated, the electrocatalytic current for CO_2_RR was measured in partially deuterated phosphate solutions at pH/pD = 5 using 2-min rotating disk CA measurements at −1.25 V vs. SCE and a rotation rate of 1600 rpm. Partially deuterated phosphate solutions were prepared by mixing appropriate amounts of the pH 5 phosphate solution and pD 5 deuterated phosphate solution. In a plot of *j*_*n*_/*j*_0_ as a function of *n*, the shape of the resulting curve is dependent on the relative sizes of *ϕ* and *Z*. *Z* > 1 suggests there is an aggregate inverse isotope effect at the *Z*-sites, and *Z* ≈ 1 suggests there are no *Z*-sites contributing to the observed kinetics (see Supplementary Information for further discussion of the *Z* parameter)^33,44^. A plot of *j*_*n*_/*j*_0_ as a function of *n* for CoPc-P4VP results in a non-linear dome-shaped response as shown in Fig. 5a, and a fit of this curve to equation (2) results in *ϕ* ≈ 0.3 and *Z* > 1. Note that *ϕ* represents the isotopic fractionation factor of the hydrogenic site involved in step (iii) in Fig. 3a, and *ϕ* ≈ 0.3 is a typical fractionation factor for transition-state hydrogen bridges corresponding to hydrogen transfer reactions of small molecules^53^. These results are consistent with a normal isotope effect at a single hydrogenic site in the rate-determining step at the active site coupled with an aggregate inverse-isotope effect from the *Z*-sites (pyridyl sites on the polymer). Note that a weak inverse-isotope effect is a somewhat common phenomena for H^+^ exchange reactions at weak bases such as pyridine^53^. The results of the CoPc-P4VP proton inventory studies support our hypothesis that proton delivery to the active site is controlled by a polymer-based proton relay mechanism. Note that in our analysis we do not take into account contributions to the overall isotope effect from the expected H-bonding interactions between the P4VP polymer and reactive CO_2_ intermediates (i.e., secondary-coordination sphere effects). This is because weak H-bonds tend to have negligible isotope effects with *ϕ* ~ 1 and therefore do not typically contribute significantly to the overall isotope effect^47^.

**Table 2:** Results of Kinetic Isotope Effects and Proton Inventory Measurements for CoPc-P4VP with different P4VP loadings

P4VP (%)	py:CoPc^a^	pyH^+^:CoPc^b^	*j*_H, D_ (mA∙cm^-2^)	TOF_CO H,D_^e^ (s^−1^)	KIE	Proton Inventory Parameters	
						*ϕ*	*Z*
0.1	190	38	(H)^c^ −2.09 ± 0.06	(H) 4.39 ± 0.16	2.3 ± 0.2	─^f^	─^f^
			(D)^d^ −0.92 ± 0.06	(D) 1.96 ± 0.13			
0.5	950	190	(H) −2.67 ± 0.10	(H) 5.81 ± 0.29	2.2 ± 0.1	0.31 ± 0.01	1.45 ± 0.03
			(D) −1.22 ± 0.05	(D) 2.59 ± 0.18			
1	1900	380	(H) −2.90 ± 0.02	(H) 6.31 ± 0.08	2.1 ± 0.1	0.29 ± 0.01	1.65 ± 0.03
			(D) −1.37 ± 0.01	(D) 3.10 ± 0.16			
2	3800	760	(H) −3.03 ± 0.09	(H) 6.73 ± 0.29	1.9 ± 0.1	─^f^	─^f^
			(D) −1.60 ± 0.06	(D) 3.36 ± 0.17			
3	5700	1140	(H) −3.17 ± 0.04	(H) 6.96 ± 0.17	1.7 ± 0.1	0.31 ± 0.01	1.89 ± 0.03
			(D) −1.82 ± 0.05	(D) 3.91 ± 0.13			

All measurements conducted at −1.25 V vs. SCE. All reported values are averages from three or more independent measurements, and all errors are given as standard deviations

^a^Ratio of py to CoPc as determined by calculating the relative amount of P4VP and CoPc drop-cast on the EPG surface

^b^Ratio of protonated pyH^+^ to CoPc assuming 20% of the pyridyl residues are protonated within the polymer at pH 5^20^

^c^Under protic condition

^d^Under deuterated condition

^e^Turnover frequencies for CO (TOF_CO_) is calculated from both the overall activity measured in rotating disk chronoamperometric (CA) steps at −1.25 V vs. SCE and the faradaic efficiencies measured in 2-h controlled potential electrolyses (CPE) (see Supplementary Information for detailed explanation)

^f^Not measured

In contrast to CoPc-P4VP, the activity of CoPc(py) shows a linear attenuation with increasing *n* confirming that without the encapsulating polymer only one hydrogenic site (the hydrogenic site involved in step (iii) in Fig. 3a) is involved in the rate-determining step of the catalytic mechanism and there is no *Z*-effect (i.e., *Z* ≈ 1). This is also consistent with the larger observed KIE = 3.1 for CoPc(py) compared to KIE = 2.1 for CoPc-P4VP, where the overall KIE is modulated by the inverse-isotope effects of the *Z*-sites. Note that CoPc(py)-P2VP with axially-coordinated pyridyls (Fig. 5a) shows a response nearly identical to that of CoPc-P4VP with identical values of *ϕ* and *Z* (Table 1). Both the parent CoPc system (Fig. 5a) and the CoPc-P2VP system (Fig. 5a) show no attenuation of activity in the proton inventory studies as expected from our KIE measurements and consistent with a rate-limiting step (i) (Fig. 3a) in the catalytic cycle for the parent system.

In order to confirm that the KIE originates from the proton channel along the partially protonated pyridyl residues rather the mass transport of protons through the polymer membrane, proton inventory studies for CoPc and CoPc(py) encapsulated in PS were performed. CoPc-PS (Fig. 5b) shows identical behavior to that of CoPc (Fig. 5a), and CoPc(py)-PS (Fig. 5b) shows identical behavior to that of CoPc(py) (Fig. 5a) for proton inventory studies. This suggests that the dome-shaped responses of CoPc-P4VP and CoPc(py)-P2VP are due to a specific proton relay effect imbued by the pyridyl moieties and not a general behavior of polymers without proton relays. Note that similar proton inventory behavior is shown in the catalytic systems in this study at other potentials investigated (see Supplementary Figs. 33–39).

### Polymer loading dependence on KIE and *Z*

Our KIE and proton inventory studies provide strong evidence that pyridyls within the polyvinylpyridine polymers act as proton relays to control proton delivery to the CoPc active sites, and the weak-inverse isotope effect from the pyridyl moieties moderates the KIE for CoPc-P4VP compared to CoPc(py). Based on these observations, we postulate that increasing the ratio of pyridyl to CoPc within the polymer-composite film should lead to an increase in the number of *Z-*sites in the film, which in turn will decrease the overall observed KIE. To test this, we investigated the proton inventory behavior and KIE for CO_2_RR by CoPc-P4VP prepared from deposition solutions with different polymer loadings and the results are summarized in Table 2. Proton inventory measurements of CoPc-P4VP (Fig. 5c, Table 2) show an increase in the *Z*-value as the polymer loading (and py-to-Co ratio) increase as expected, and the measured KIE values decrease as the polymer loading increases (Fig. 5d). These results are consistent with our previous observations regarding the existence of pyridyl-based proton relays with weak inverse isotope effects in the polyvinylpyridine polymers. Note that the CO_2_RR activity slightly increases with increasing P4VP loading. We postulate that this may be due to increased CO_2_ partitioning within largely-hydrophobic polymer layer which may lead to higher overall catalytic activity^20,22^.

## Discussion

We believe the experimental techniques applied in and the mechanistic insights derived from this work can serve as a model for understanding the catalytic mechanisms of related heterogeneous electrocatalysts. For example, previous studies of CO_2_ reduction with CoPc adsorbed onto graphitic carbon show non-selective CO_2_ reduction to CO with ε_CO_ ranging from ~40% to ~60% with appreciable competitive H_2_ evolution^19,21,22,56^, which is consistent with this study. However, recent reports show that CoPc adsorbed onto highly-oxygenated carbon nanotubes (CNTs) synthesized from high-temperature calcination of carbon-precursors in air selectively reduces CO_2_ with ε_CO_ ~ 80−90% at optimized potential, pH, and loading conditions^40,56,57^. It has been postulated that the π-π interactions between the CNTs and the macrocyclic CoPc complexes may explain the increased activity of CoPc and related systems when absorbed onto CNTs^40,56,57^. We propose that an additional reason for the increased activity of CoPc adsorbed onto CNTs may be axial-coordination of impurities in the CNTs structure, such as oxide- and hydroxyl-defect sites, with the adsorbed CoPc. These proposed axial interactions in the CoPc-CNT system are analogous to the axial-coordination of pyridine and P4VP to CoPc in our studies. Note that similar increases in activity for O_2_ reduction by macrocyclic Co and Fe complexes adsorbed onto defect-rich carbon supports has been previously observed^58–62^, and was largely attributed to axial-coordination of the metal complexes to organic functional groups on the carbon surfaces^59–62^. While directly probing the nature of the CoPc-CNTs interactions is beyond the scope of our current study, we suggest that similar electrochemical KIE measurements to those conducted here can be used as a tool to determine the nature of the rate-determining step of CO_2_ reduction by CoPc adsorbed onto CNTs and thereby determine the nature of the CoPc-CNTs interactions.

Perhaps one of the most-promising CO_2_RR materials mechanistically related to the CoPc and CoPc-P4VP systems is MNC materials—extended graphitic structures with discrete M- N_4_ porphyrin-like active sites^14^. Studies exploring the mechanism of low-overpotential CO_2_ reduction at M-N_4_ active sites suggest that rate-determining step is a decoupled proton-electron transfer event forming an M-CO_2_H adduct via a process similar to step (i) and subsequent intramolecular H^+^ transfer in step (ii) shown in Fig. 3a^12,15^. In addition, CO production has been shown to be pH independent at the M-N_4_ site^15^, which is consistent with our observation that CoPc and CoPc-P2VP operate with the same TOF_CO_ despite the increase in local pH near the Co active site in CoPc-P2VP as evidenced by the decrease in competitive H_2_ evolution for CoPc-P2VP compared to CoPc. The correlation between the mechanistic insights provided in previous studies for the MNC materials and our mechanistic observations for CoPc-P4VP and related systems suggest that the polymer-encapsulated CoPc materials may be a useful and easily-tunable model system that, in future studies, can be leveraged to provide further insight into the activity, selectivity and mechanism of CO_2_ reduction by heterogeneous MNC materials.

We have investigated the electrochemical CO_2_RR mechanism for CoPc encapsulated in a coordinating polymer using a combination of KIE measurements and proton inventory studies. Specifically, KIE studies suggest that axial-coordination of pyridyl/pyridine to CoPc to form a putative five-coordinate species changes the rate-determining step of the catalytic mechanism from a CO_2_-binding step (step (i), Fig. 3a) in the case of CoPc to a subsequent protonation step (step (iii), Fig. 3a) in the case of the five-coordinate species. The axially-coordinated pyridine/pyridyl can be either a discrete ligand (CoPc(py), CoPc(py)-P2VP, CoPc(py)-PS) or be attached to an encapsulating polymer (CoPc-P4VP). Moreover, our proton inventory studies strongly suggest that proton delivery to the CoPc active sites in the polyvinylpyridine-encapsulated systems is controlled by a polymer-based proton relay mechanism involving the pyridyl moieties. Our work here provides a strategy to modulate the catalytic activity of this class of catalyst-polymer composite systems by (1) controlling the extent of axial-coordination to the catalyst center and (2) controlling the fractional protonation of the polymer to modulate the nature and extent of the proton relays in the encapsulating polymer. The mechanistic insights for the CoPc-P4VP and related systems introduced in this work reinforce the findings of previous studies of catalytic mechanism at M-N_4_ active sites in heterogeneous MNC materials. The systems and experimental techniques developed in this work will serve as a useful model for further probing catalytic activity and mechanisms in future MNCs and polymer-encapsulated catalyst materials which will facilitate the development of new, more-active electrocatalytic systems for selective CO_2_ reduction.

## Methods

### Electrolyte solution preparation and pH measurements

All pH measurements were conducted with a Fisher Scientific Accumet AB200 pH meter with an Accumet pH/ATC Epoxy Body Combination Electrode calibrated with a three-point calibration curve at pH = 4.01, 7.00, and 10.01. For estimating pD, measurements were conducted in deuterated solvents using the pH meter and the pD was calculated by the following equation: pD = pH_meter reading + _0.40^63,64^. pH 5 phosphate solutions were prepared from 0.1 M NaH_2_PO_4_ solutions adjusted to pH 5 by the addition of aqueous 1 M NaOH. pD 5 deuterated phosphate solutions were prepared by titrating 1.189 g D_3_PO_4_ (85 wt % solution) with 1.025 g NaOD (40 wt % solution) in ~100 mL D_2_O to produce a 0.1 M NaD_2_PO_4_ solution in D_2_O, and then titrated with 1 M NaOD D_2_O solution. Partially deuterated phosphate solutions were prepared by mixing appropriate amounts of the pH 5 phosphate solution and pD 5 deuterated phosphate solution.

### Preparation of modified electrodes

All deposition solutions were prepared from DMF solutions containing 0.05 mM CoPc. The deposition solutions for polymer-encapsulated CoPc were prepared by dissolving the desired amount of polymer in the 0.05 mM CoPc/DMF solution. For the deposition of CoPc-P4VP films, the deposition solution contained 0.1–3% w/v of P4VP in DMF solution (detailed preparation conditions are provided in Supplementary Methods). In the case of CoPc-P4VP films, the py:Co ratio was determined by calculating the relative amount of pyridyl groups in P4VP and CoPc drop-cast on the EPG surface.

For the deposition solution of CoPc(py), a mixture of pyridine and DMF solution (19:1 DMF/pyridine) was used as the solvent in place of DMF. To confirm that the deposited film from the above method was indeed CoPc(py), we also independently synthesized five-coordinate CoPc(py) and confirmed it using elemental analysis (Supplementary Table 7). Drop-cast films prepared using our traditional method and the synthesized CoPc(py) showed analogous KIE and proton inventory results (see Supplementary Fig. 40 and Supplementary Table 8), suggesting the prepared films are identical.

Prior to modification, 5 mm diameter edge plane graphite (EPG) disk electrodes (3.81 mm EPG disk encapsulated in epoxy, 0.114 cm^2^ effective surface area, Pine Research Instrumentation) were manually polished with 600 grit SiC grinding paper (Buehler CarbiMet) followed by sonication in ultrapure water for ~1 min. Modified working electrodes were prepared by first drop-casting 5 μL deposition solution onto EPG electrode. The disks electrodes were then placed in a drying oven at ~70 °C for ~15 min to allow the solvent to evaporate.

### Electrochemical measurements

Electrochemical measurements were conducted using a Bio-Logic SP200 potentiostat/galvanostat, and data were recorded using the Bio-Logic EC-Lab software package. Reference electrodes were commercial saturated calomel electrodes (SCE, CH-Instruments) externally referenced to ferrocenecarboxylic acid in 0.2 M phosphate buffer at pH 7 (0.284 V vs. SCE)^65^, and auxiliary electrodes were carbon rods (99.999%, Strem Chemicals Inc.). Working electrodes were the modified EPG electrodes described previously. In all cases, the working electrode was separated from the auxiliary electrode by a Nafion membrane. Unless otherwise noted, all electrochemical measurements were conducted at least three times with independently prepared electrodes, all values reported are the averages of these repetitions, and all reported errors are standard deviations.

For rotating disk CA step measurements, the modified EPG working electrodes were mounted in a Pine Research Instrumentation E6-series change disk rotating disk electrode (RDE) assembly attached to an MSR rotator. CA measurements were conducted at 1600 rpm with 2-min potential steps from −1.00 V to −1.25 V vs. SCE at 0.05 V increments. The 1600 rpm rotation rate was meant to ensure steady-state delivery of substrate to our surface to allow for accurate comparisons of catalytic rates. Note that 1600 rpm does not imply kinetically-limiting conditions—mass transport to catalyst sites in non-uniform catalyst-polymer composite films is not governed by simple Koutecký-Levich kinetics^66–68^. Rotating disk CA measurements were conducted in a custom two-compartment glass cell (Supplementary Fig. 41). The first compartment held the rotating disk working electrode and reference electrode in ~30 mL solution, and the second compartment held the auxiliary electrode in ~15 mL solution. The two compartments were separated by a Nafion cation exchange membrane. Both compartments were sparged with CO_2_ for ~30 min prior to each set of measurements, and the headspace was blanketed with CO_2_ during the measurements. The CO_2_ used was first saturated with electrolyte solution by bubbling through a gas washing bottle filled with the same electrolyte solution used in the cell to minimize electrolyte evaporation in the cell during the course of the measurements. IR drop was compensated at 85% through positive feedback using the Bio-Logic EC-Lab software. In general, our electrochemical cell for CA measurement had Ru = ∼100 Ω in pH 5 or pD 5 phosphate solution.

CPE were conducted at room temperature in two custom, gas-tight, two-chamber U-cells (Supplementary Fig. 42). The modified working electrode was held in a RDE internal hardware kit (Pine Research Instrumentation) and mounted into a custom PEEK sleeve. For the electrolysis measurements, the main chamber held the working electrode and an SCE reference electrode in ~25 mL of electrolyte, and the total headspace in the main chamber was measured individually after each experiment, ~25 mL, by measuring the amount of water needed to refill the main chamber. The auxiliary chamber held the auxiliary carbon rod electrode in 15 mL electrolyte. The two chambers were separated with a Nafion cation exchange membrane. Prior to each experiment, both chambers were sparged with CO_2_ for ~30 min and then the main chamber was sealed under CO_2_ atmosphere. The uncompensated resistance of the cell was measured with a single-point high-frequency impedance measurement. In general, our electrochemical cell for CPE had Ru = ∼200 Ω in pH 5 or pD 5 phosphate solution.

### Product detection and quantification

After CPE, gaseous and liquid samples were collected and analyzed using gas chromatography (GC) and high-performance liquid chromatography (HPLC), respectively. For gaseous samples, analysis was conducted using a Thermo Scientific Trace 1310 GC system with two analyzer channels for the detection of H_2_ and C1-C2 products. A Pressure-Lok gas-tight syringe (10 mL, Valco VICI Precision Sampling, Inc.) was used to collect 5 mL aliquots from the main-chamber headspace of the cell, and each aliquot was injected directly into the 3 mL sample loop. Using a custom valve system, column configuration, and method provided by Thermo Scientific, gases were separated such that H_2_ was detected on the first channel using an Ar carrier gas and thermal conductivity detector (TCD), and all other gases were detected on the second channel using a He carrier gas and a TCD. The GC system was calibrated using calibration gas mixtures (SCOTTY Specialty Gas) at H_2_ = 0.02, 0.05, 0.5, and 1% v/v, and CO = 0.02, 0.05, 0.5, 1, and 7% v/v. An example chromatograph of a calibration mixture containing 0.05% H_2_, 0.05% CO, and 99.9% N_2_, is shown in Supplementary Fig. 43. Chromatographs were analyzed using the Chromeleon Console WorkStation software.

For liquid samples, 1 mL aliquots of post-electrolysis solutions were analyzed for liquid products such as formic acid using a Thermo Scientific UltiMate 3000 HPLC system equipped with a refractive index detector (RFD), a 5 cm Thermo Scientific™ HyperREZ™ XP Carbohydrate H^+^ LC guard column and a 30 cm Thermo Scientific™ HyperREZ™ XP Carbohydrate H^+^ LC analytical column in series using a 5 mM H_2_SO_4_ aqueous mobile phase at a constant temperature of 50 °C. The detection limit of the HPLC for formic acid was determined to be 0.1 mM. In general, no formic acid was observed in the electrolyte solution after the electrolyses.

Faradaic efficiencies (*ɛ*) were determined by dividing the moles of each product detected by the total moles of electrons calculated from the amount of charge passed during the CPE as described in Eq. (4):

4 $$\epsilon = \frac{{\frac{{V_{\mathrm{HS}}}}{V} \times C \times 2F}}{Q}$$

Here, *V*_HS_ is the volume of the headspace in the main chamber of the cell (mL), *V* is the molar volume of gas at 25 °C and 1.0 atm (24.5 L mol^−1^), *C* is the volume percent of product detected by GC (%), *F* is the Faraday constant (C mol^−1^), and *Q* is the charge passed during the CPE measurement (C).

## Supplementary information

Supplementary Information

Source Data

## Data Availability

The authors declare that all data supporting the findings of this study are available within the paper and its Supplementary Information. The source data underlying Figs. 4, 5 and Tables 1, 2, Supplementary Figs. 1–40, 43, and Supplementary Tables 1–6, 8 are provided as a Source Data file.
