# Supplementary material for: Modulating the mechanism of electrocatalytic CO2 reduction by cobalt phthalocyanine through polymer coordination and encapsulation
Source: Nat Commun. 2019 Apr 11;10:1683. doi: 10.1038/s41467-019-09626-8 (PMC6459859; doi:10.1038/s41467-019-09626-8)
Supplement: Supplementary file 1 — Supplementary Information [file 41467_2019_9626_MOESM1_ESM.pdf]

Modulating the mechanism of electrocatalytic CO_2 reduction by cobalt phthalocyanine through polymer coordination and encapsulation

Supplementary Information

Yingshuo Liu and Charles C. L. McCrory*

Department of Chemistry, University of Michigan, Ann Arbor, Michigan 48109, United States.

Supplementary Figures

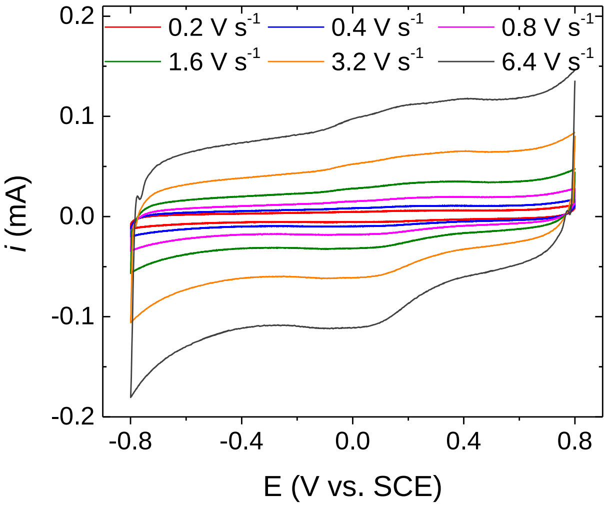

Supplementary Figure 1. Cyclic voltammograms of CoPc on EPG in pH 5 phosphate solution recorded at potentials positive of the catalytic current at different scan rates under N_2 atmosphere. The redox couple at $E_{1/2}$ = 0.18 V is assigned to the $[CoPc]^+/[CoPc]$ couple. The broad peaks are consistent with π-π stacking and aggregation of the CoPc molecules

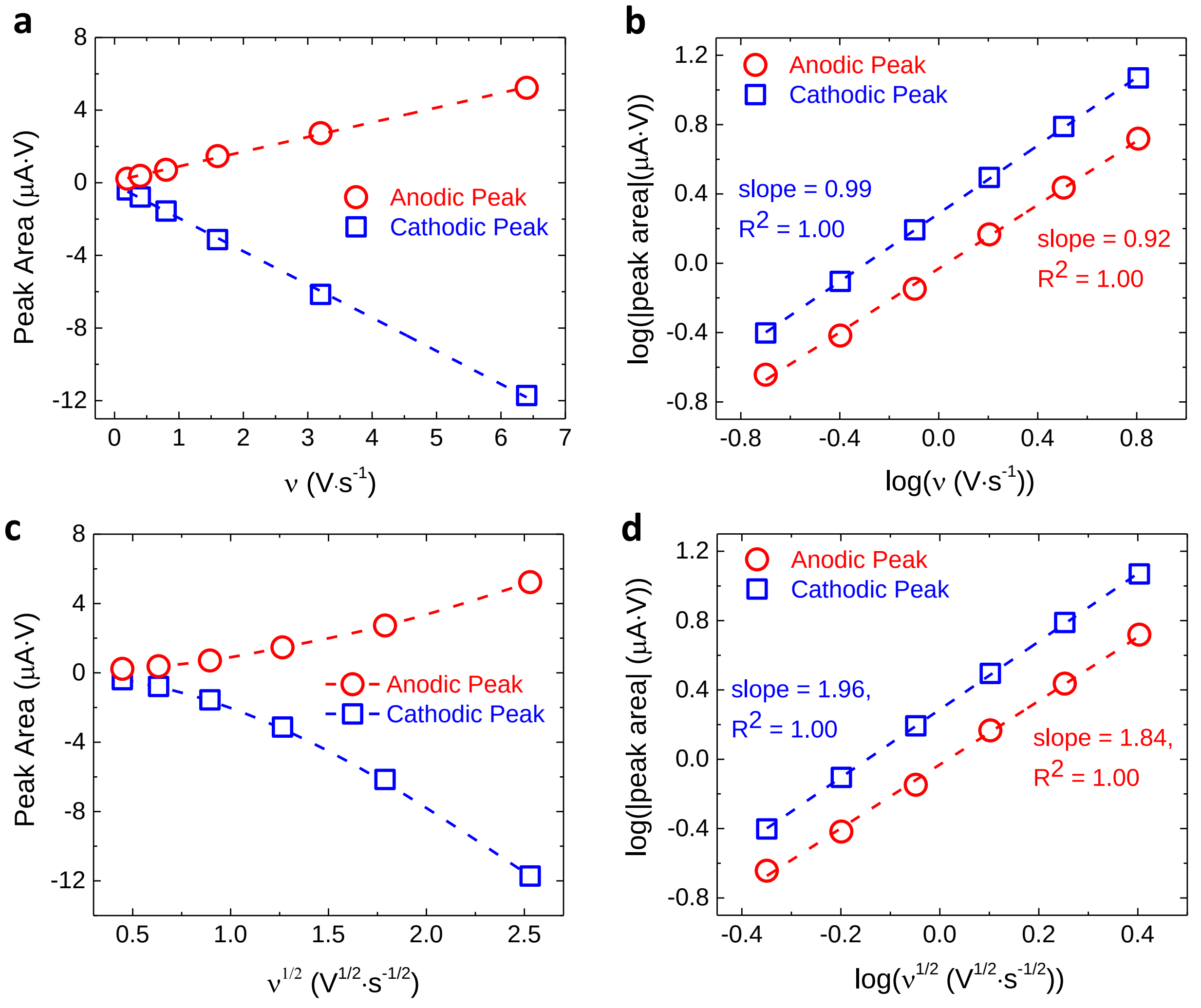

Supplementary Figure 2. Scan Rate Dependence Study of CoPc on EPG

a, Plots of peak area vs. scan rate for the $[CoPc]^+$/[CoPc] peaks in CoPc are linear, which is consistent with electron transfer to a surface-immobilized species.

b, Plots of log(|peak area|) vs. log(scan rate) for the $[CoPc]^+$/[CoPc] peaks in CoPc have slopes ~ 1, which is consistent with a 1st order dependence on scan rate as expected for a surface-immobilized species.

c, Plots of peak area vs. $(\text{scan rate})^{1/2}$ for the $[CoPc]^+$/[CoPc] peaks in CoPc are non-linear which is not consistent with electron transfer to a diffusing species in solution.

d, Plots of log(|peak area|) vs. $\log((\text{scan rate})^{1/2})$ for the $[CoPc]^+$/[CoPc] peaks in CoPc have slopes ~ 2, which is inconsistent with electron transfer to a diffusing species in solution and instead is consistent with a surface-immobilized species.

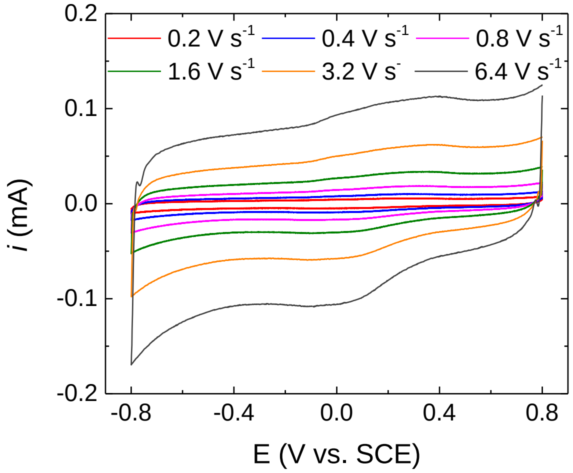

Supplementary Figure 3. Cyclic voltammograms of CoPc(py) on EPG in pH 5 phosphate solution recorded at potentials positive of the catalytic current at different scan rates under N_2 atmosphere. The redox couple at $E_{1/2}$ = 0.18 V is assigned to the $[CoPc]^+/[CoPc]$ couple. The broad peaks are consistent with π-π stacking and aggregation of the CoPc molecules.

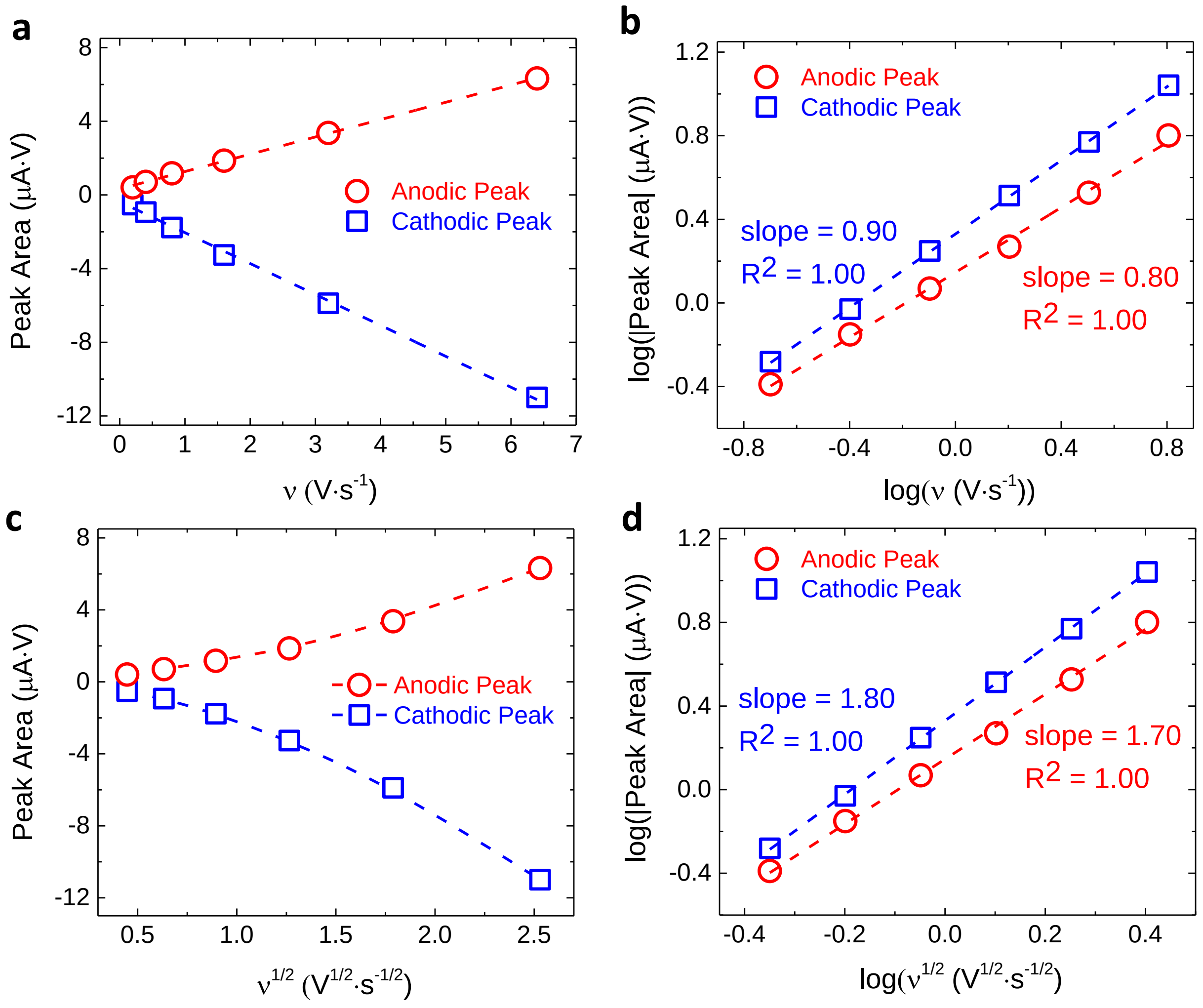

Supplementary Figure 4. Scan Rate Dependence Study of CoPc(py) on EPG

a, Plots of peak area vs. scan rate for the $[CoPc]^+$/[CoPc] peaks in CoPc(py) are linear, which is consistent with electron transfer to a surface-immobilized species.

b, Plots of log(|peak area|) vs. log(scan rate) for the $[CoPc]^+$/[CoPc] peaks in CoPc(py) have slopes ~ 1, which is consistent with a 1st order dependence on scan rate as expected for a surface-immobilized species.

c, Plots of peak area vs. $(\text{scan rate})^{1/2}$ for the $[CoPc]^+$/[CoPc] peaks in CoPc(py) are non-linear which is not consistent with electron transfer to a diffusing species in solution.

d, Plots of log(|peak area|) vs. log($(\text{scan rate})^{1/2}$) for the $[CoPc]^+$/[CoPc] peaks in CoPc(py) have slopes ~ 2, which is inconsistent with electron transfer to a diffusing species in solution and instead is consistent with a surface-immobilized species.

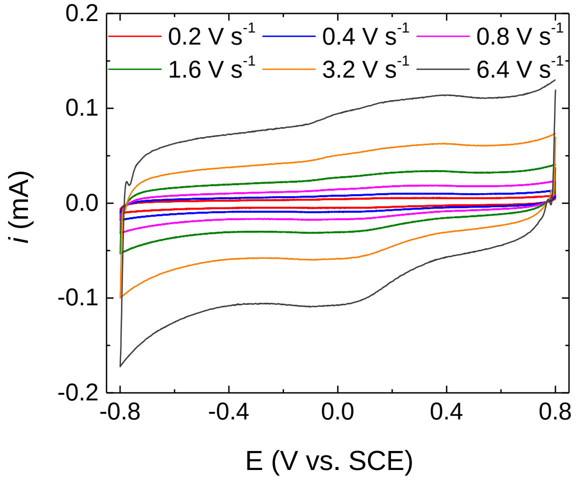

Supplementary Figure 5. Cyclic voltammograms of CoPc-P4VP on EPG in pH 5 phosphate solution recorded at potentials positive of the catalytic current at different scan rates under N_2 atmosphere. The redox couple at $E_{1/2}$ = 0.18 V is assigned to the $[CoPc]^+/[CoPc]$ couple. The broad peaks are consistent with π-π stacking and aggregation of the CoPc molecules.

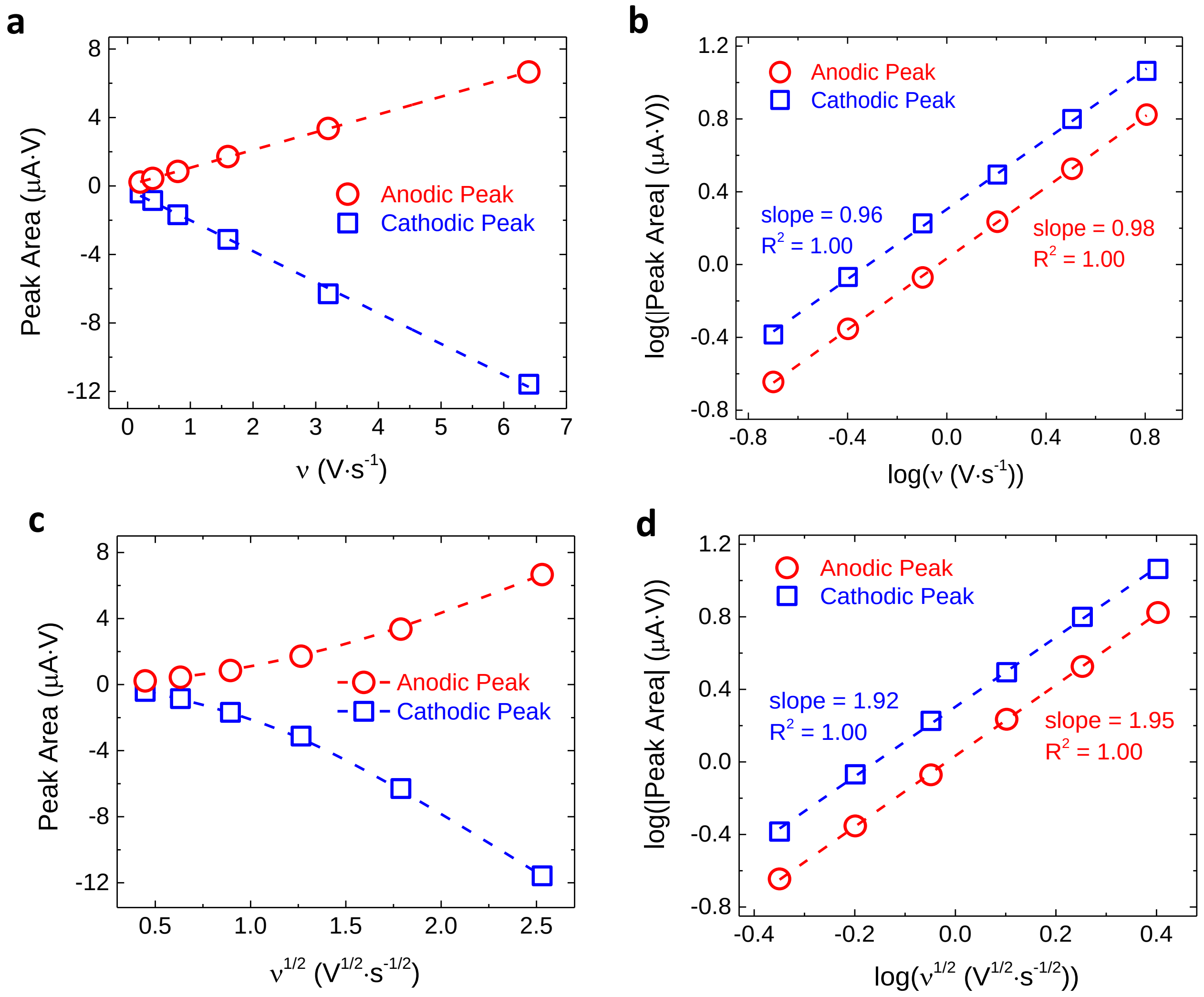

Supplementary Figure 6. Scan Rate Dependence Study of CoPc-P4VP on EPG

a, Plots of peak area vs. scan rate for the $[CoPc]^+$/[CoPc] peaks in CoPc-P4VP are linear, which is consistent with electron transfer to a surface-immobilized species.

b, Plots of log(|peak area|) vs. log(scan rate) for the $[CoPc]^+$/[CoPc] peaks in CoPc-P4VP have slopes ~ 1, which is consistent with a 1st order dependence on scan rate as expected for a surface-immobilized species.

c, Plots of peak area vs. (scan rate)$^{1/2}$ for the $[CoPc]^+$/[CoPc] peaks in CoPc-P4VP are non-linear which is not consistent with electron transfer to a diffusing species in solution.

d, Plots of log(|peak area|) vs. log((scan rate)$^{1/2}$) for the $[CoPc]^+$/[CoPc] peaks in CoPc-P4VP have slopes ~ 2, which is inconsistent with electron transfer to a diffusing species in solution and instead is consistent with a surface-immobilized species.

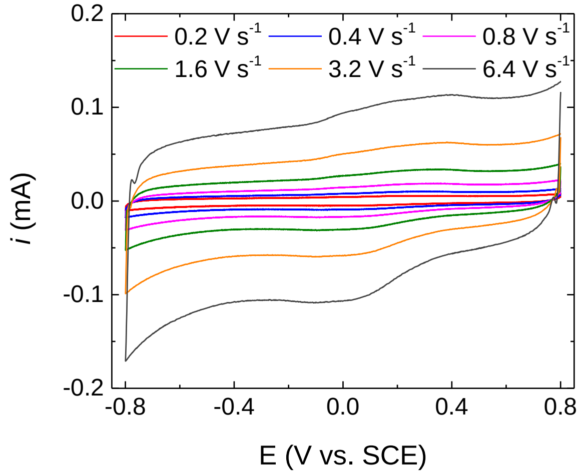

Supplementary Figure 7. Cyclic voltammograms of CoPc-P2VP on EPG in pH 5 phosphate solution recorded at potentials positive of the catalytic current at different scan rates under N_2 atmosphere. The redox couple at $E_{1/2}$ = 0.18 V is assigned to the $[CoPc]^+/[CoPc]$ couple. The broad peaks are consistent with π-π stacking and aggregation of the CoPc molecules.

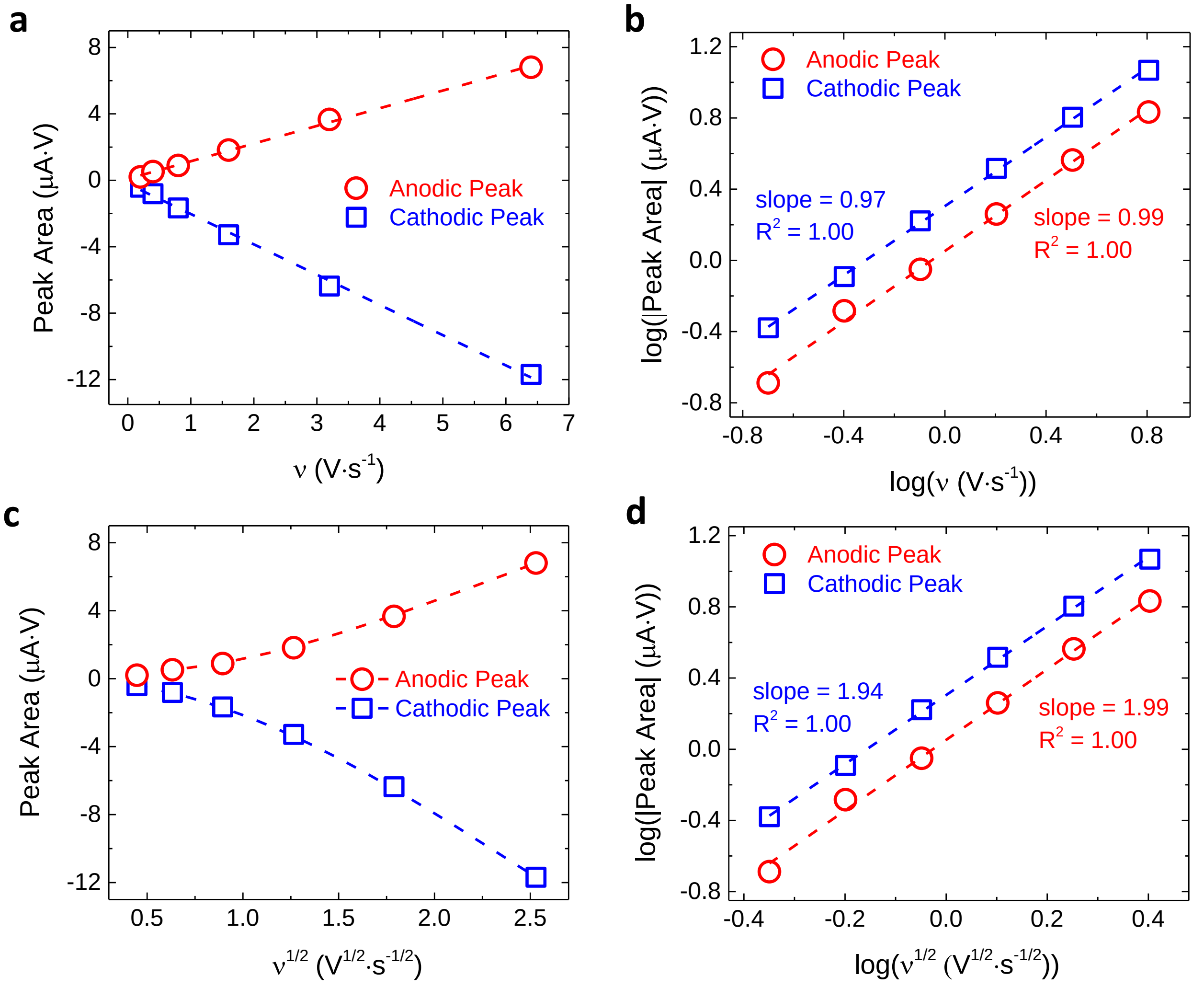

Supplementary Figure 8. Scan Rate Dependence Study of CoPc-P2VP on EPG

a, Plots of peak area vs. scan rate for the $[CoPc]^+$/[CoPc] peaks in CoPc-P2VP are linear, which is consistent with electron transfer to a surface-immobilized species.

b, Plots of log(|peak area|) vs. log(scan rate) for the $[CoPc]^+$/[CoPc] peaks in CoPc-P2VP have slopes ~ 1, which is consistent with a 1st order dependence on scan rate as expected for a surface-immobilized species.

c, Plots of peak area vs. (scan rate)$^{1/2}$ for the $[CoPc]^+$/[CoPc] peaks in CoPc-P2VP are non-linear which is not consistent with electron transfer to a diffusing species in solution.

d, Plots of log(|peak area|) vs. log((scan rate)$^{1/2}$) for the $[CoPc]^+$/[CoPc] peaks in CoPc-P2VP have slopes ~ 2, which is inconsistent with electron transfer to a diffusing species in solution and instead is consistent with a surface-immobilized species.

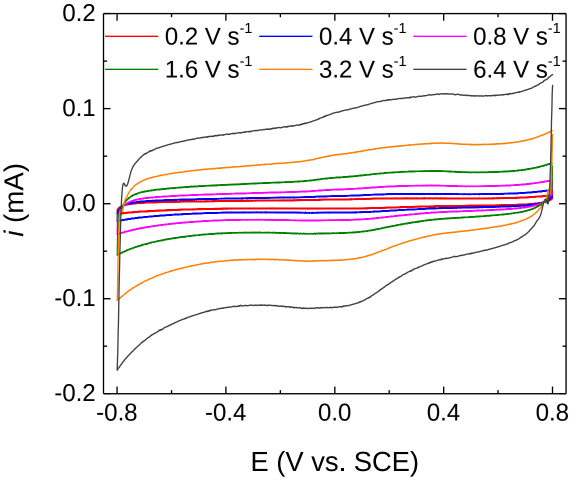

Supplementary Figure 9. Cyclic voltammograms of CoPc(py)-P2VP on EPG in pH 5 phosphate solution recorded at potentials positive of the catalytic current at different scan rates under N_2 atmosphere. The redox couple at $E_{1/2}$ = 0.18 V is assigned to the $[CoPc]^+$/[CoPc] couple. The broad peaks are consistent with π-π stacking and aggregation of the CoPc molecules.

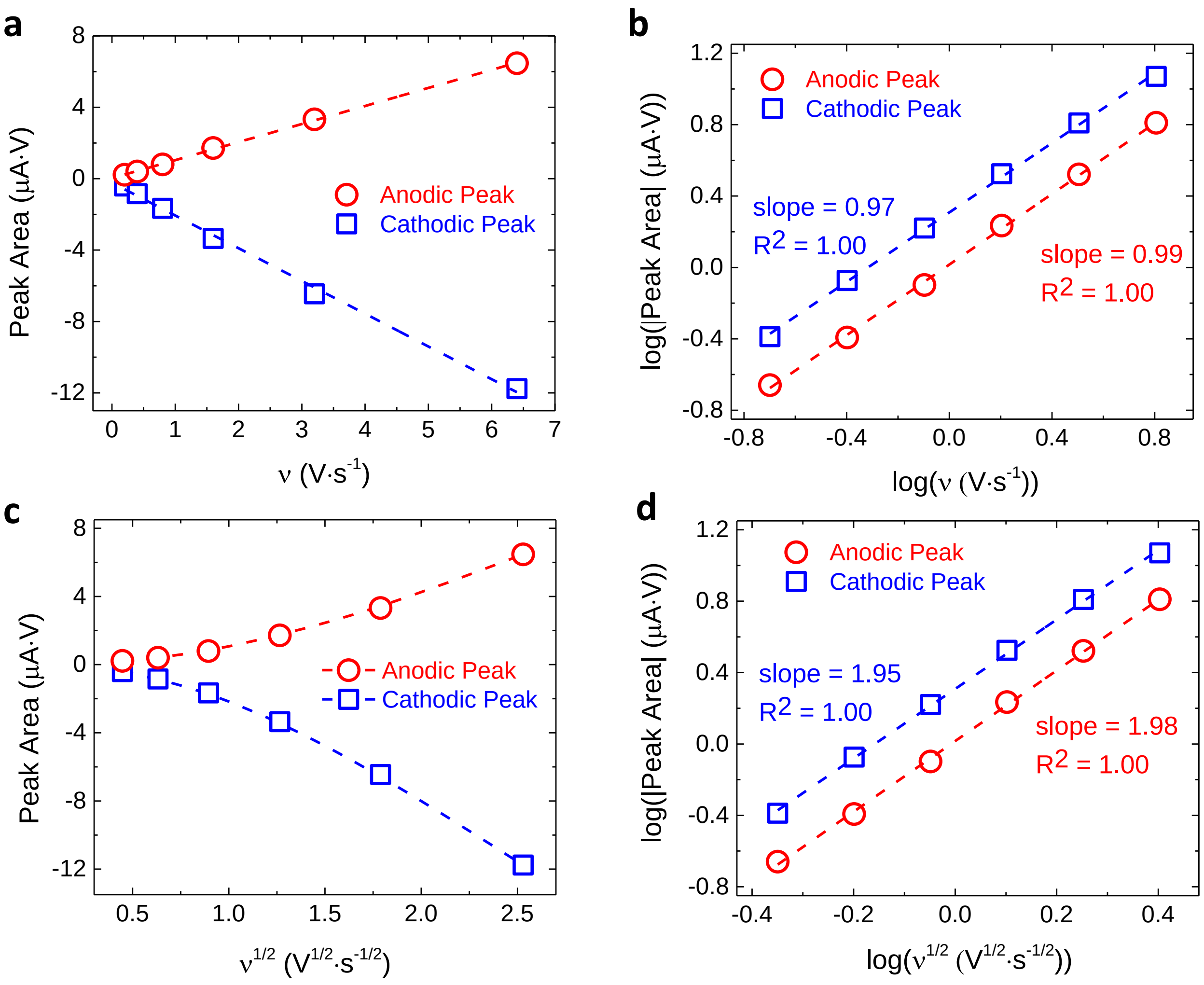

Supplementary Figure 10. Scan Rate Dependence Study of CoPc(py)-P2VP on EPG

a, Plots of peak area vs. scan rate for the $[CoPc]^+$/[CoPc] peaks in CoPc(py)-P2VP are linear, which is consistent with electron transfer to a surface-immobilized species.

b, Plots of log(|peak area|) vs. log(scan rate) for the $[CoPc]^+$/[CoPc] peaks in CoPc(py)-P2VP have slopes ~ 1, which is consistent with a 1st order dependence on scan rate as expected for a surface-immobilized species.

c, Plots of peak area vs. (scan rate)$^{1/2}$ for the $[CoPc]^+$/[CoPc] peaks in CoPc(py)-P2VP are non-linear which is not consistent with electron transfer to a diffusing species in solution.

d, Plots of log(|peak area|) vs. log((scan rate)$^{1/2}$) for the $[CoPc]^+$/[CoPc] peaks in CoPc(py)-P2VP have slopes ~ 2, which is inconsistent with electron transfer to a diffusing species in solution and instead is consistent with a surface-immobilized species.

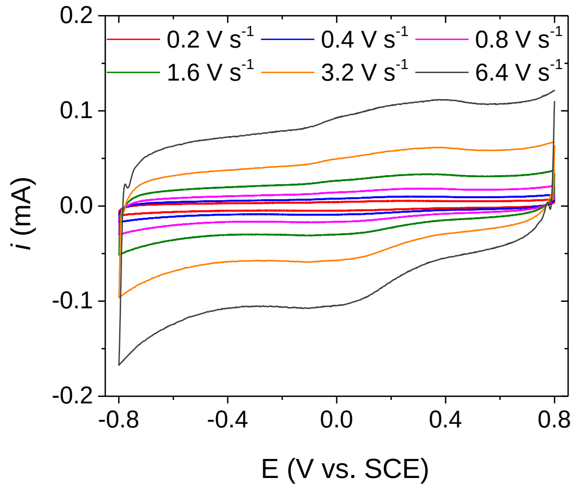

Supplementary Figure 11. Cyclic voltammograms of CoPc-PS on EPG in pH 5 phosphate solution recorded at potentials positive of the catalytic current at different scan rates under N_2 atmosphere. The redox couple at $E_{1/2}$ = 0.18 V is assigned to the $[CoPc]^+$/[CoPc] couple. The broad peaks are consistent with π-π stacking and aggregation of the CoPc molecules.

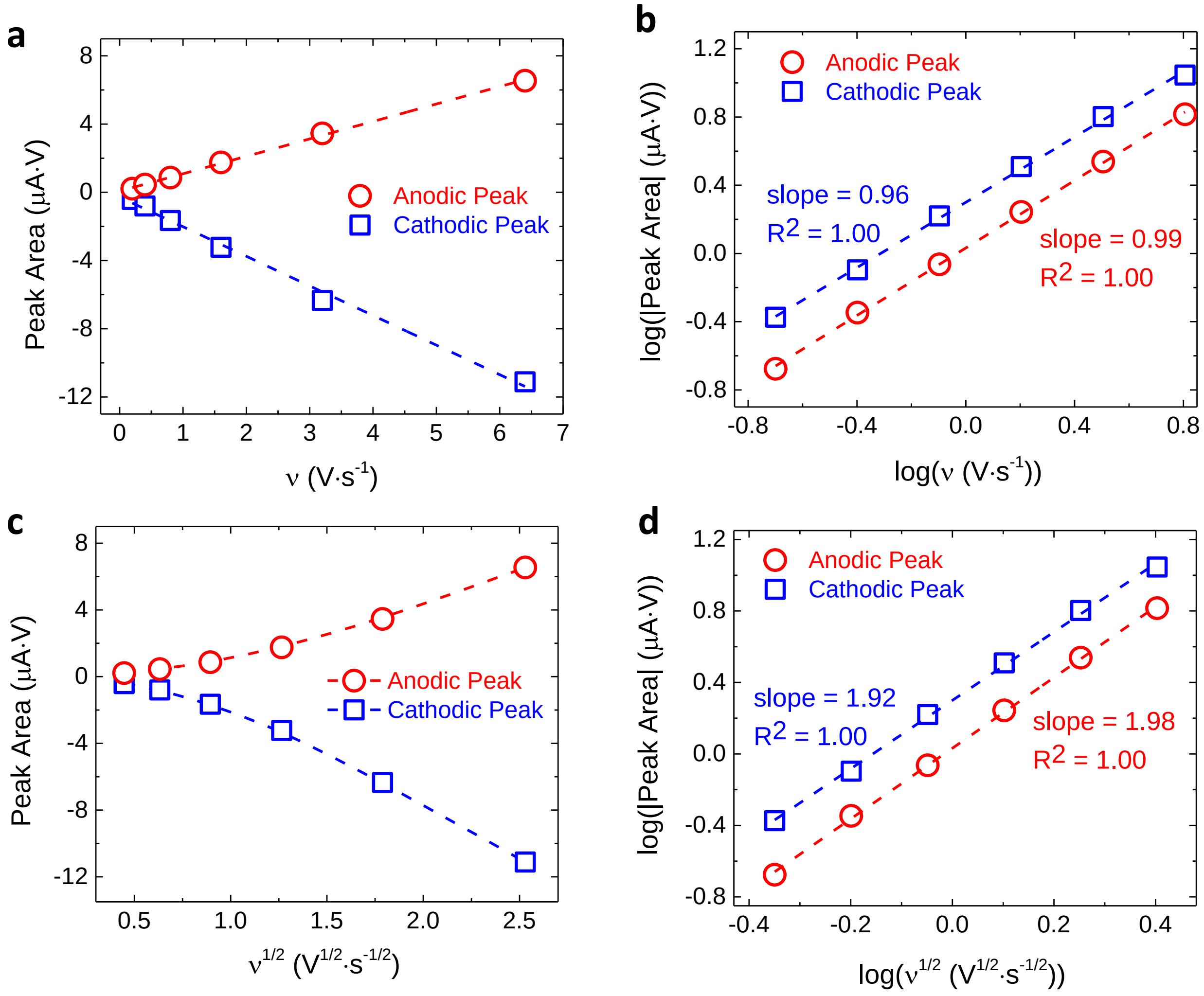

Supplementary Figure 12. Scan Rate Dependence Study of CoPc-PS on EPG

a, Plots of peak area vs. scan rate for the $[CoPc]^+$/[CoPc] peaks in CoPc-PS are linear, which is consistent with electron transfer to a surface-immobilized species.

b, Plots of log(|peak area|) vs. log(scan rate) for the $[CoPc]^+$/[CoPc] peaks in CoPc-PS have slopes ~ 1, which is consistent with a 1st order dependence on scan rate as expected for a surface-immobilized species.

c, Plots of peak area vs. (scan rate)$^{1/2}$ for the $[CoPc]^+$/[CoPc] peaks in CoPc-PS are non-linear which is not consistent with electron transfer to a diffusing species in solution.

d, Plots of log(|peak area|) vs. log((scan rate)$^{1/2}$) for the $[CoPc]^+$/[CoPc] peaks in CoPc-PS have slopes ~ 2, which is inconsistent with electron transfer to a diffusing species in solution and instead is consistent with a surface-immobilized species.

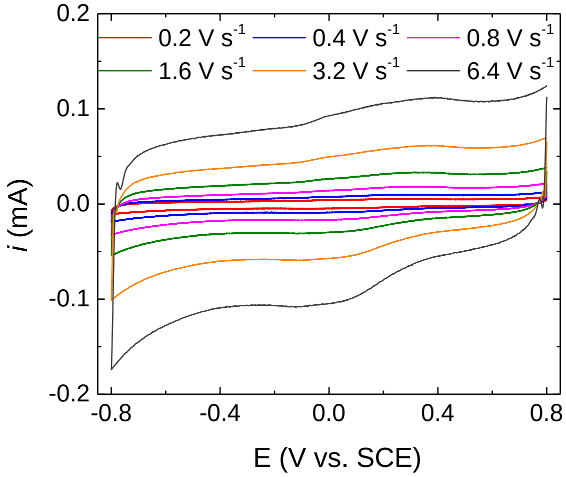

Supplementary Figure 13. Cyclic voltammograms of CoPc(py)-PS on EPG in pH 5 phosphate solution recorded at potentials positive of the catalytic current at different scan rates under N_2 atmosphere. The redox couple at $E_{1/2}$ = 0.18 V is assigned to the $[CoPc]^+$/[CoPc] couple. The broad peaks are consistent with π-π stacking and aggregation of the CoPc molecules.

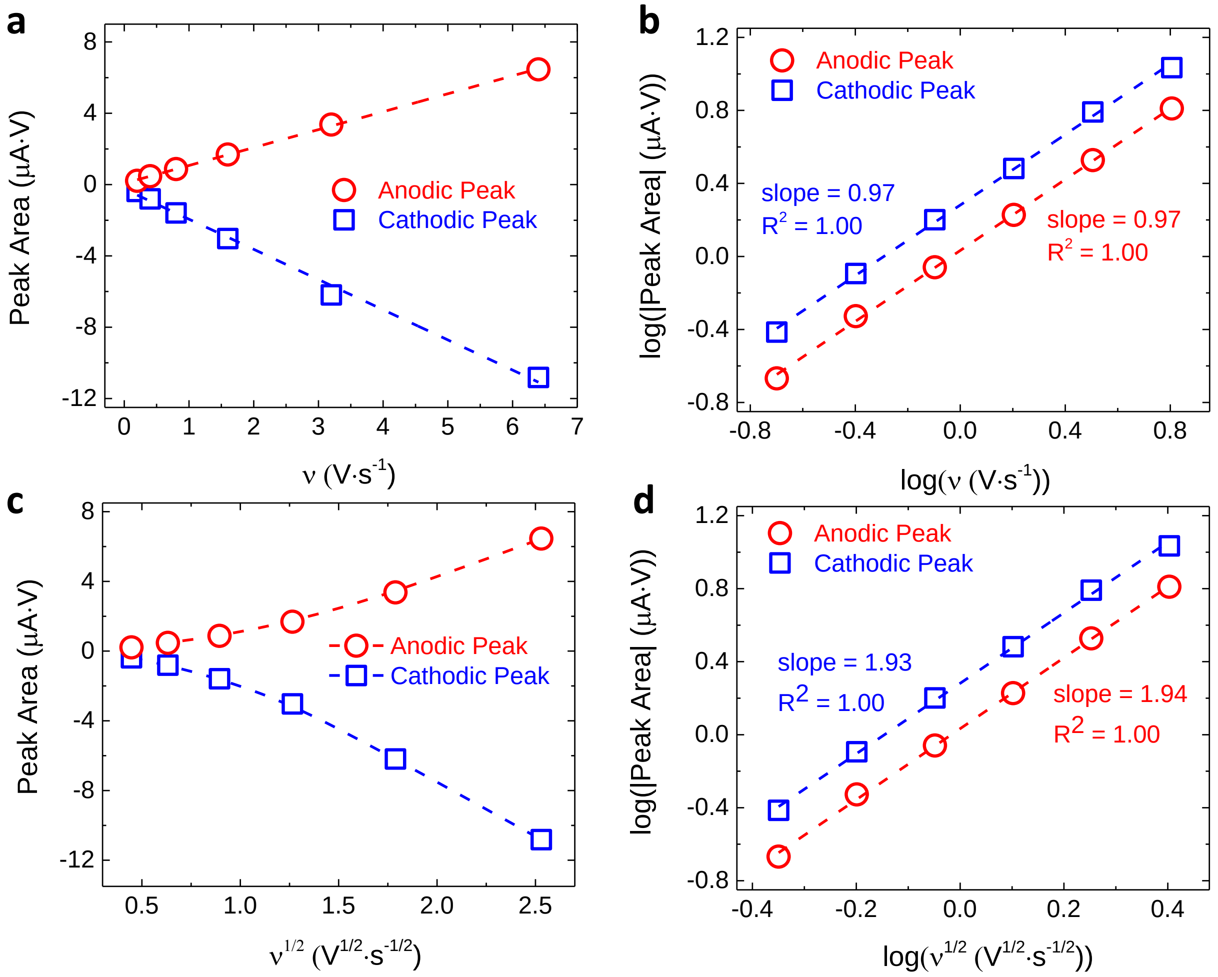

Supplementary Figure 14. Scan Rate Dependence Study of CoPc(py)-PS on EPG

a, Plots of peak area vs. scan rate for the $[CoPc]^+$/[CoPc] peaks in CoPc(py)-PS are linear, which is consistent with electron transfer to a surface-immobilized species.

b, Plots of log(|peak area|) vs. log(scan rate) for the $[CoPc]^+$/[CoPc] peaks in CoPc(py)-PS have slopes ~ 1, which is consistent with a 1st order dependence on scan rate as expected for a surface-immobilized species.

c, Plots of peak area vs. (scan rate)$^{1/2}$ for the $[CoPc]^+$/[CoPc] peaks in CoPc(py)-PS are non-linear which is not consistent with electron transfer to a diffusing species in solution.

d, Plots of log(|peak area|) vs. log((scan rate)$^{1/2}$) for the $[CoPc]^+$/[CoPc] peaks in CoPc(py)-PS have slopes ~ 2, which is inconsistent with electron transfer to a diffusing species in solution and instead is consistent with a surface-immobilized species.

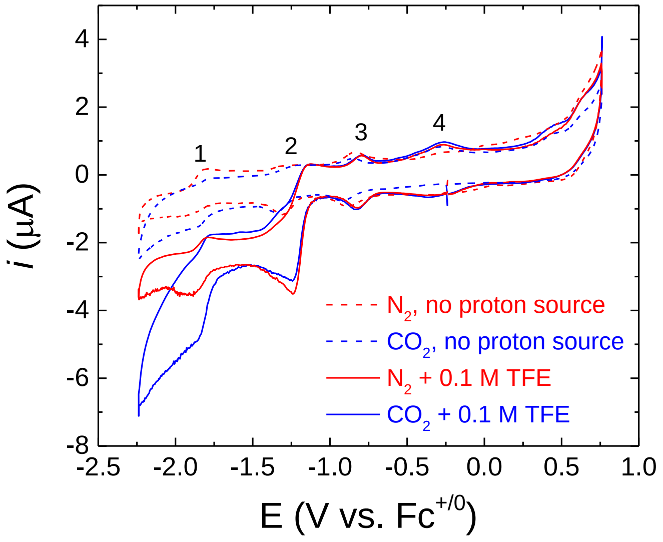

Supplementary Figure 15. Cyclic voltammogram (CV) of 0.2 mM CoPc in DMSO with 0.1 M nBu_4NPF_6 under N_2 and CO_2 without any proton source added, and in the presence of 0.1 M TFE. Conditions: scan rate: 50 mV/s; working electrode: glassy carbon working electrode; reference electrode: Ag/$AgNO_3$ (1 mM); counter electrode: Pt wire. Note that all CVs have been IR compensated. In the absence of CO_2 and proton source, four reversible peaks (labeled 1-4) are observed at $E_{1/2}$ = -1.80 V, -1.22 V, -0.75 V, and -0.25 V vs. $Fc^{+/0}$, respectively. We tentatively assign these peaks to the following redox couples: 1) $[Co^{I}Pc]^{2-}/[Co^{I}Pc]^{3-}$, 2) $[Co^{I}Pc]^{-}/[Co^{I}Pc]^{2-}$, 3) $[Co^{II}Pc]^{0}/[Co^{I}Pc]^{-}$ and 4) $[Co^{III}Pc]^{+}/[Co^{II}Pc]^{0}$, which is consistent with previously reported electrochemical studies of CoPc in organic solutions.[1-3] The onset potential of the electrocatalytic HER occurs near peak 2 at ~ -1.1 V vs. $Fc^{+/0}$, and the onset potential of the electrocatalytic CO_2 reduction occurs near peak 1 at ~ -1.75 V vs. $Fc^{+/0}$.

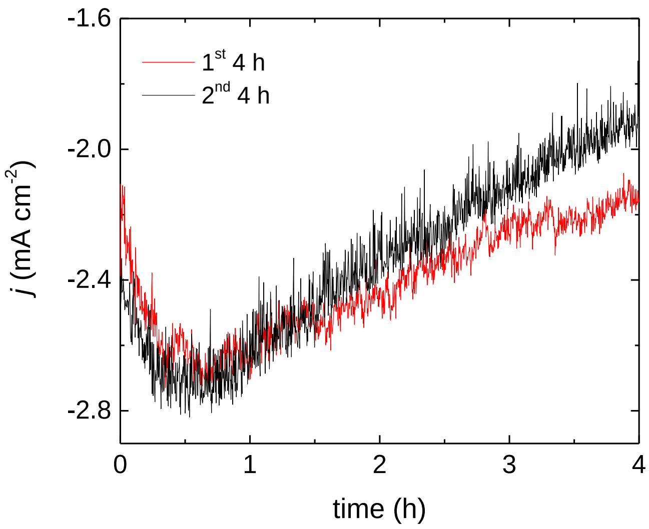

Supplementary Figure 16. Representative 4-h controlled potential electrolyses (CPE) for CoPc-P4VP at -1.25 V vs. SCE, the 2nd 4 h CPE is conducted after re-saturate the same electrolyte with CO_2 with the same catalyst. See Supplementary Table 3 for the results from CPE.

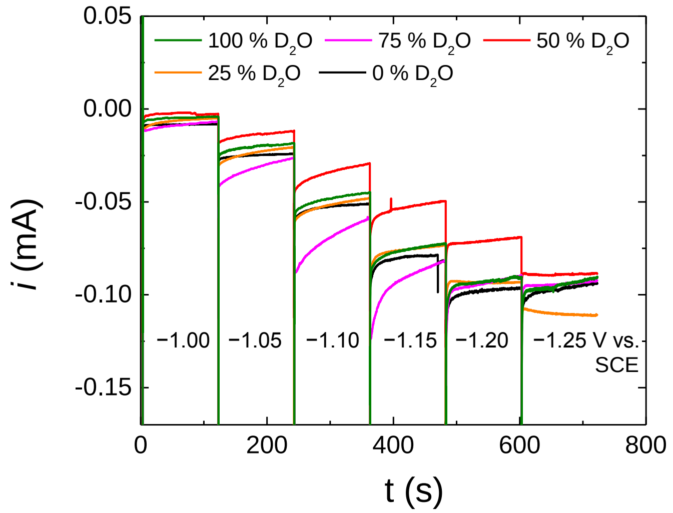

Supplementary Figure 17. Representative rotating disk chronoamperometric (CA) step measurements conducted at 1600 rpm with 2-min potential steps from -1.00 V to -1.25 V vs. SCE at 0.05 V increments for CoPc.

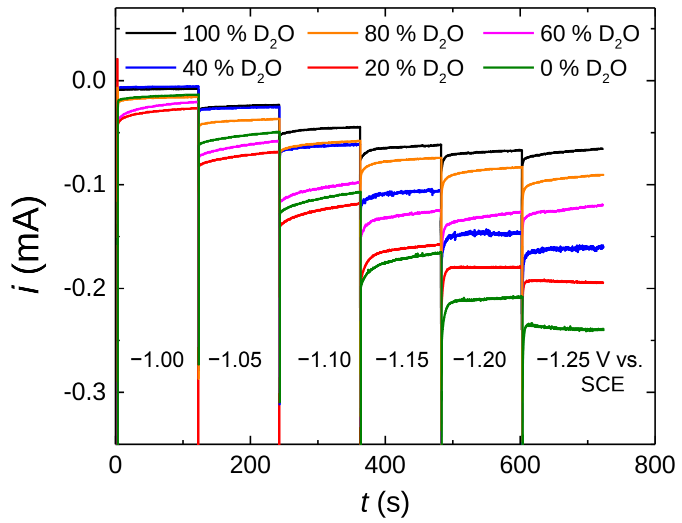

Supplementary Figure 18. Representative rotating disk chronoamperometric (CA) step measurements conducted at 1600 rpm with 2-min potential steps from -1.00 V to -1.25 V vs. SCE at 0.05 V increments for CoPc(py).

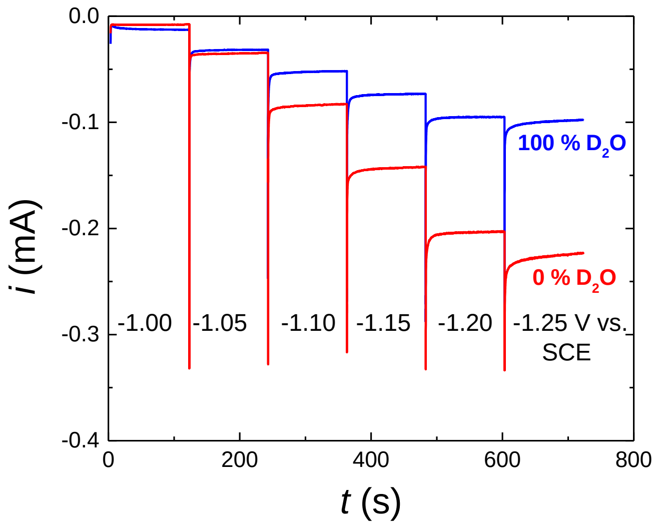

Supplementary Figure 19. Representative rotating disk chronoamperometric (CA) step measurements conducted at 1600 rpm with 2-min potential steps from -1.00 V to -1.25 V vs. SCE at 0.05 V increments for CoPc-0.1 %P4VP.

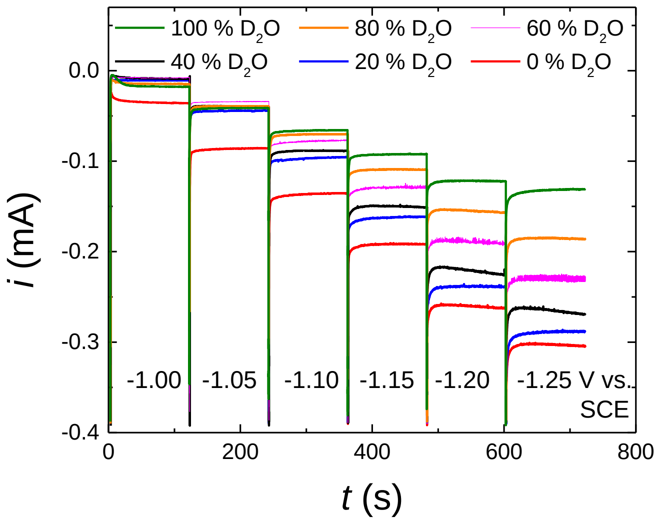

Supplementary Figure 20. Representative rotating disk chronoamperometric (CA) step measurements conducted at 1600 rpm with 2-min potential steps from -1.00 V to -1.25 V vs. SCE at 0.05 V increments for CoPc-0.5 %P4VP.

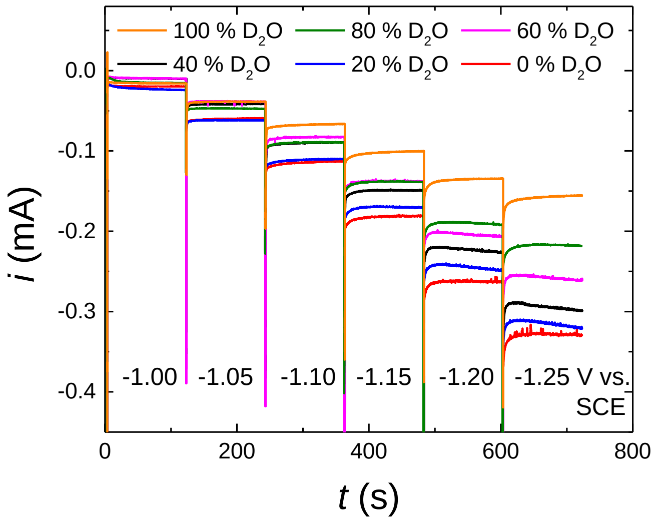

Supplementary Figure 21. Representative rotating disk chronoamperometric (CA) step measurements conducted at 1600 rpm with 2-min potential steps from -1.00 V to -1.25 V vs. SCE at 0.05 V increments for CoPc-1 %P4VP.

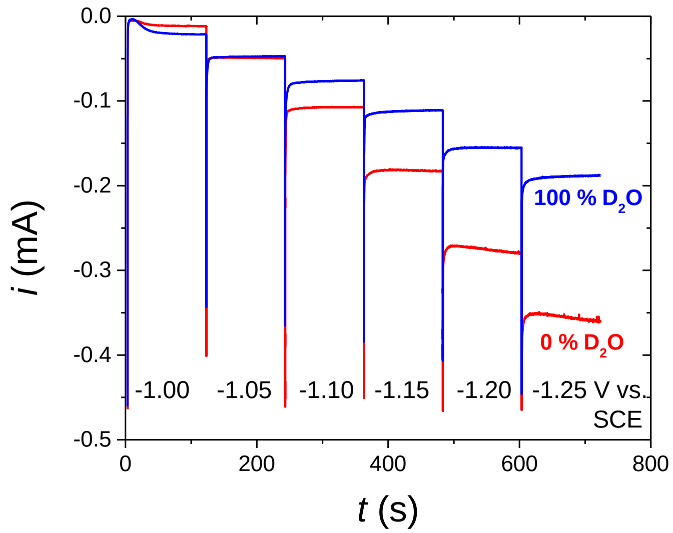

Supplementary Figure 22. Representative rotating disk chronoamperometric (CA) step measurements conducted at 1600 rpm with 2-min potential steps from -1.00 V to -1.25 V vs. SCE at 0.05 V increments for CoPc-2 %P4VP.

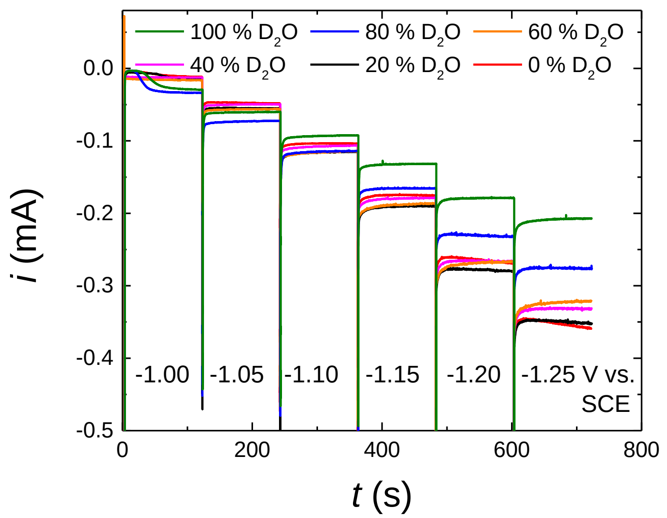

Supplementary Figure 23. Representative rotating disk chronoamperometric (CA) step measurements conducted at 1600 rpm with 2-min potential steps from -1.00 V to -1.25 V vs. SCE at 0.05 V increments for CoPc-3 %P4VP.

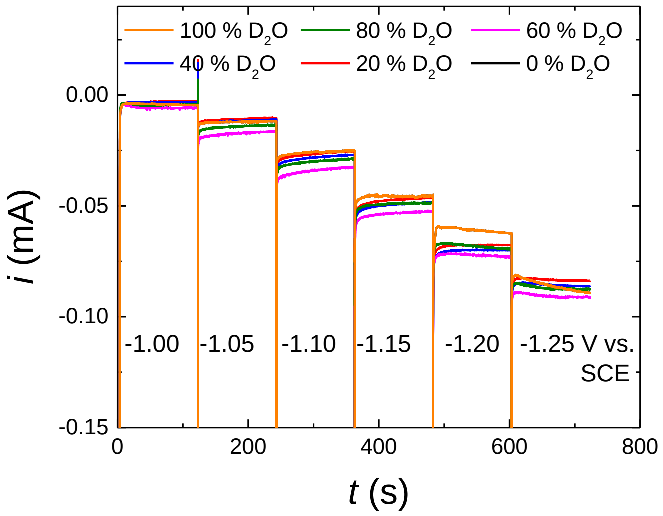

Supplementary Figure 24. Representative rotating disk chronoamperometric (CA) step measurements conducted at 1600 rpm with 2-min potential steps from -1.00 V to -1.25 V vs. SCE at 0.05 V increments for CoPc-P2VP.

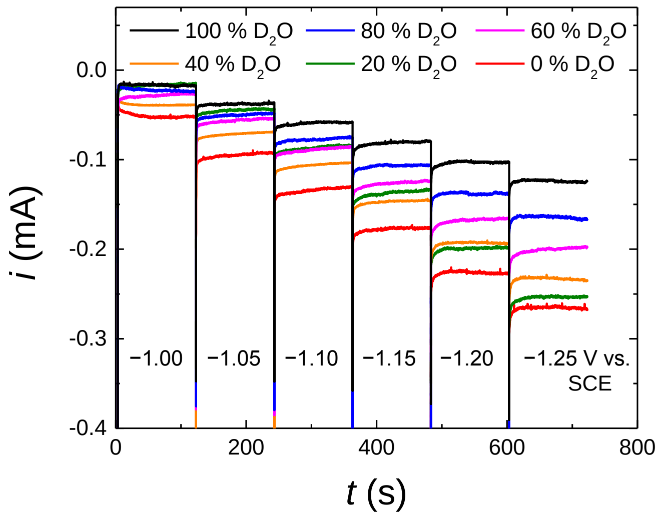

Supplementary Figure 25. Representative rotating disk chronoamperometric (CA) step measurements conducted at 1600 rpm with 2-min potential steps from -1.00 V to -1.25 V vs. SCE at 0.05 V increments for CoPc(py)-P2VP.

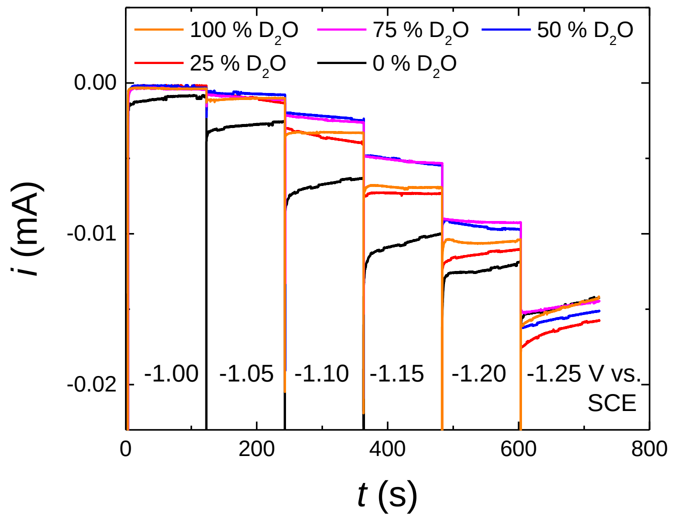

Supplementary Figure 26. Representative rotating disk chronoamperometric (CA) step measurements conducted at 1600 rpm with 2-min potential steps from -1.00 V to -1.25 V vs. SCE at 0.05 V increments for CoPc-PS.

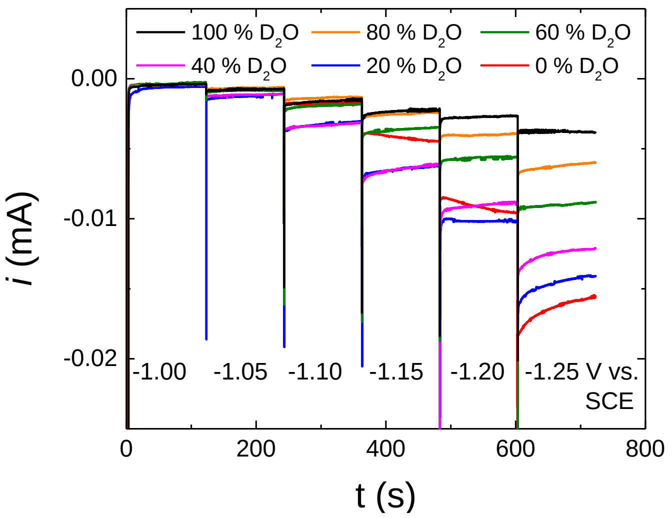

Supplementary Figure 27. Representative rotating disk chronoamperometric (CA) step measurements conducted at 1600 rpm with 2-min potential steps from -1.00 V to -1.25 V vs. SCE at 0.05 V increments for CoPc(py)-PS.

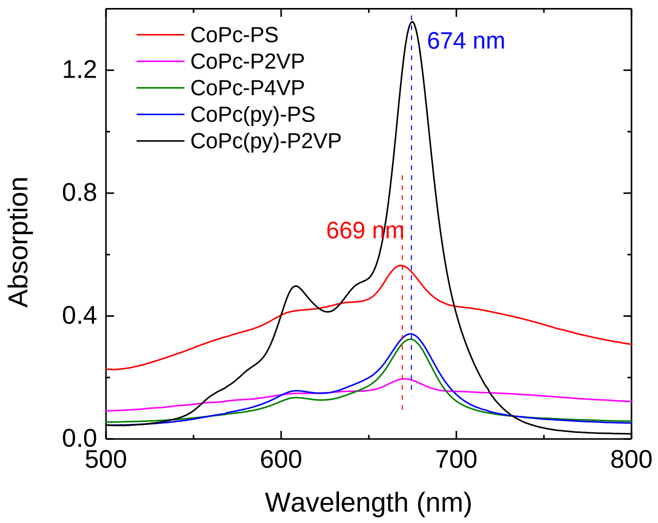

Supplementary Figure 28. UV-vis spectrum of CoPc-PS, CoPc-P2VP, CoPc-P4VP, CoPc(py)-PS, and CoPc(py)-P2VP films coated on glass slide. The Q band of CoPc in PS and P2VP near 669 nm is red shifted to 674 nm in UV-vis absorption spectrum of CoPc in P4VP, and CoPc(py) in PS and P2VP films, suggesting the formation of the axial coordination of CoPc in CoPc-P4VP, CoPc(py)-PS, CoPc(py)-P2VP samples.

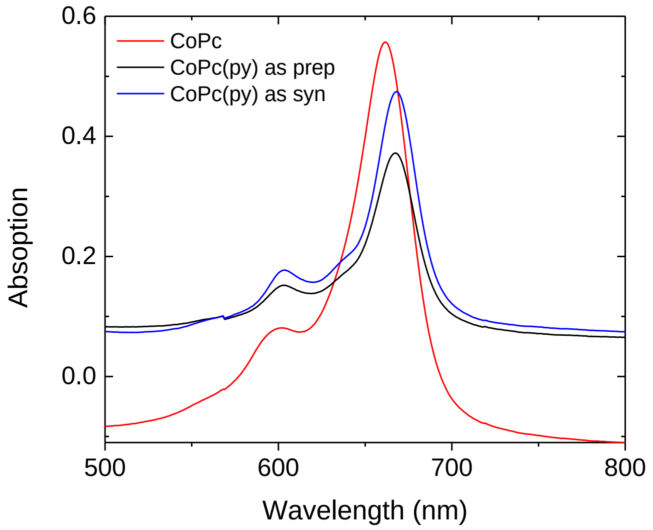

Supplementary Figure 29. UV-vis spectrum of 0.01 mM CoPc solution, 0.01 mM CoPc(py) solution as prepared, and 0.01 mM CoPc(py) solution as synthesized. Red shifted Q band is exhibited in the UV-vis spectrum of CoPc(py) as prepared and CoPc(py) as synthesized solutions by about 5 nm compared to that of CoPc, suggesting the formation of the axial coordination of CoPc in CoPc(py) solutions.

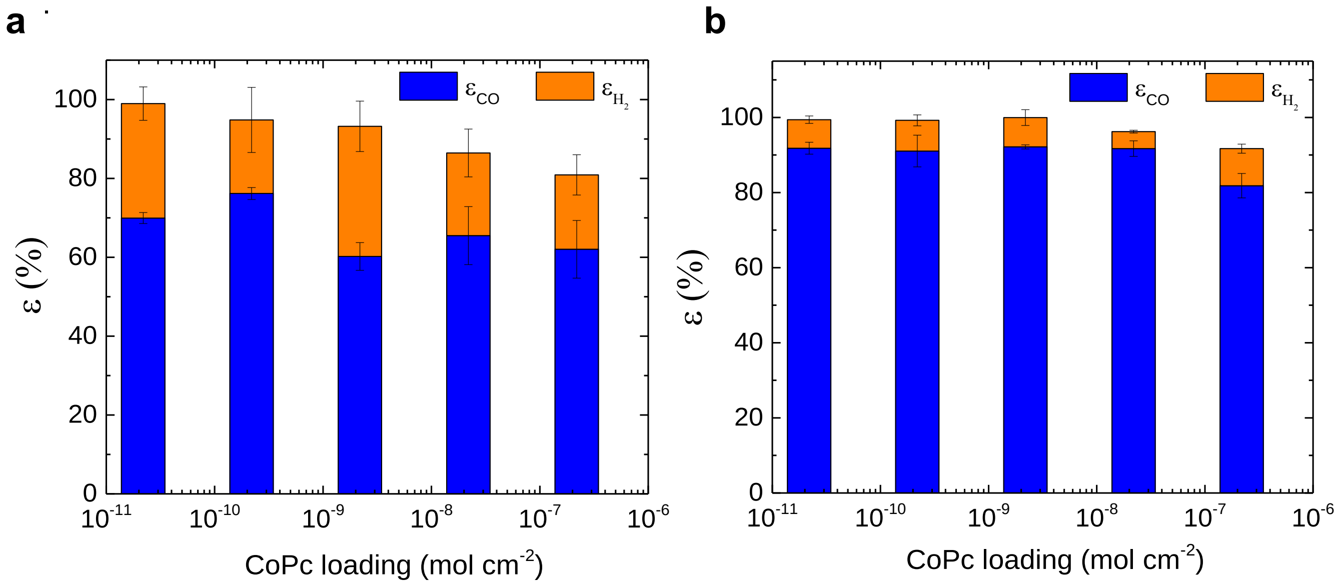

Supplementary Figure 30. Faradaic efficiencies (ε) obtained from 2-h controlled potential electrolyses (CPE) at -1.25 V vs. SCE for **a,** CoPc catalysts, and **b,** CoPc-P4VP catalysts at different CoPc loadings in CO_2 saturated pH 5 sodium phosphate electrolyte under CO_2 atmosphere. All reported values are averages from 3 independent measurements, and all errors are given as standard deviations.

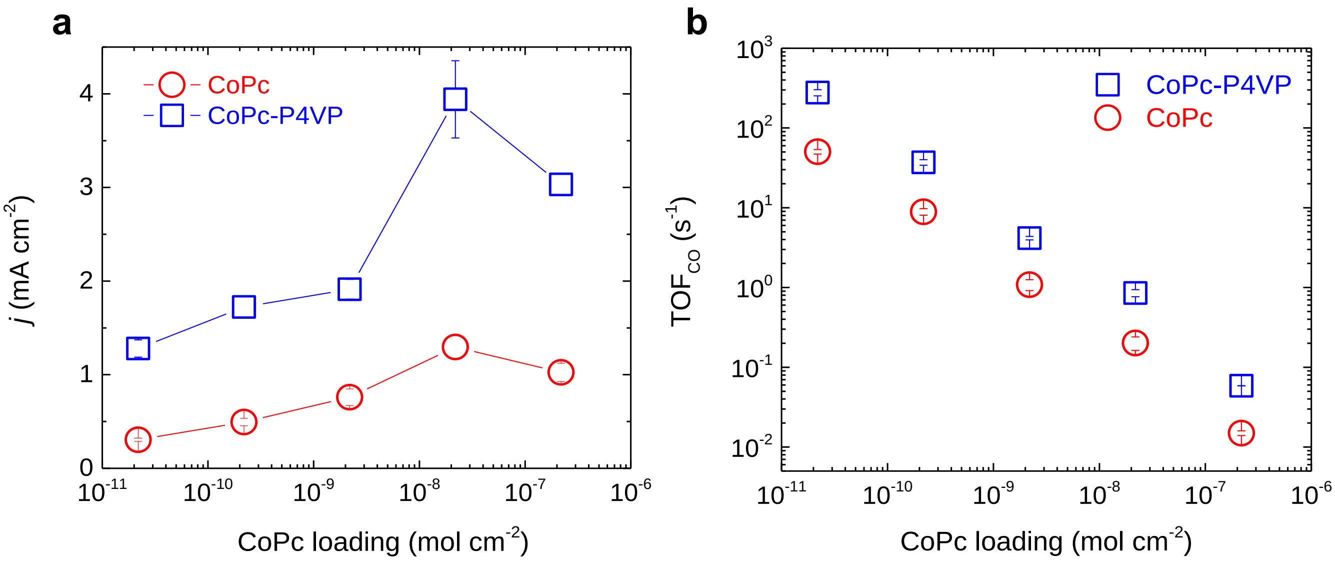

Supplementary Figure 31. CO_2 reduction results of 2-h controlled potential electrolyses (CPE) at different CoPc loadings a, Overall current densities (*j*), and **b,** Turnover frequencies for CO (TOF_{CO}) obtained from 2-h CPE at -1.25 V vs. SCE for CoPc and CoPc-P4VP catalysts at different CoPc loadings in CO_2 saturated pH 5 sodium phosphate electrolyte under CO_2 atmosphere. All reported values are averages from 3 independent measurements, and all errors are given as standard deviations.

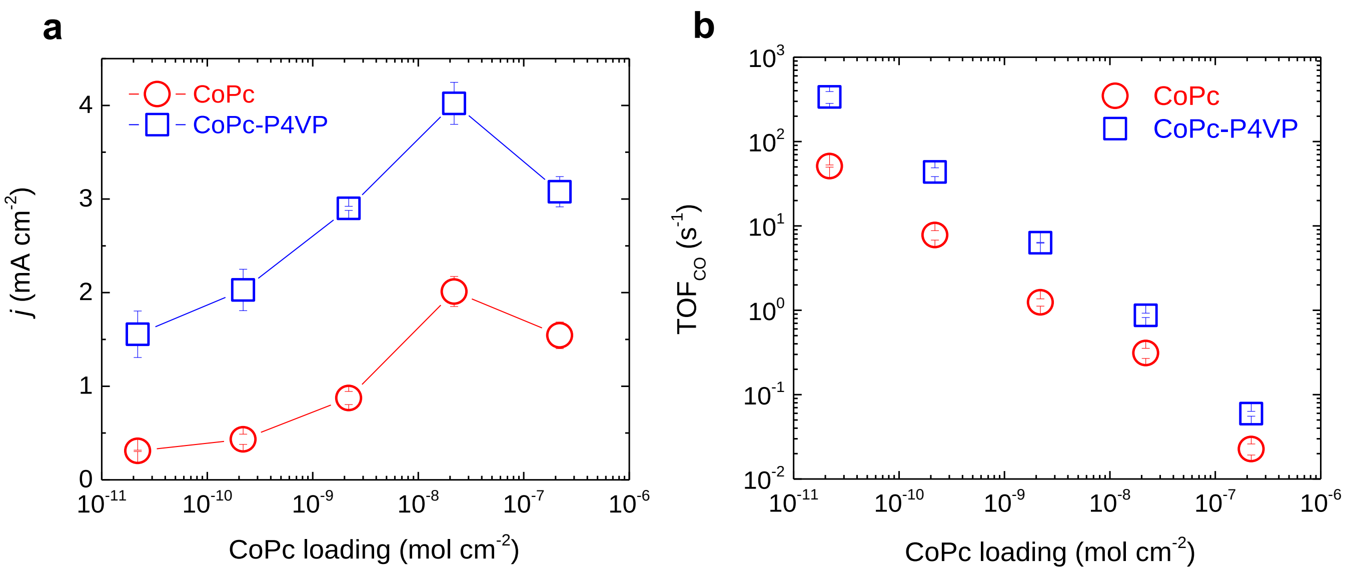

Supplementary Figure 32. CO_2 reduction results of rotating disk chronoamperometric (CA) step measurements at different CoPc loadings a, Overall current densities (*j*), and **b,** Turnover frequencies for CO (TOF_{CO}) obtained from rotating disk CA step measurements at -1.25 V vs. SCE for CoPc and CoPc-P4VP catalysts at different CoPc loadings in CO_2 saturated pH 5 sodium phosphate electrolyte under CO_2 atmosphere (See Supplementary Information for detailed TOF calculations). All reported values are averages from 3 independent measurements, and all errors are given as standard deviations.

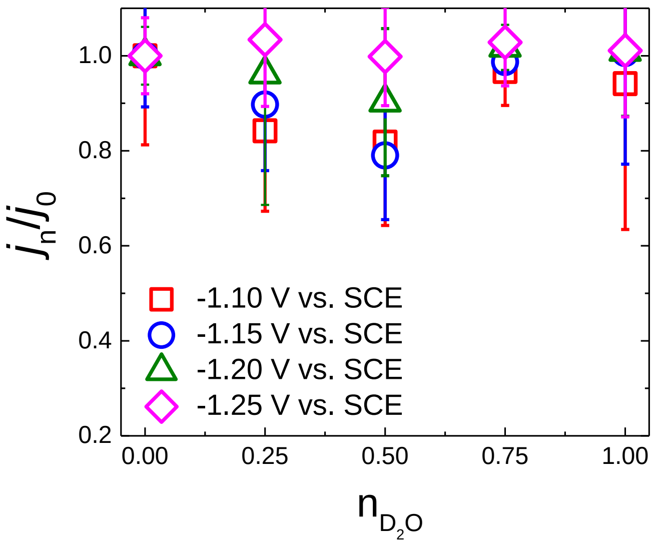

Supplementary Figure 33. Proton inventory measurements of CO_2 reduction by CoPc at different potentials based on chronoamperometric (CA) steps. Note: Data at −1.00 V and −1.05 V vs. SCE were not included because minimal catalytic current is observed at those potentials. All reported values are averages from 3 or more independent measurements, all errors are given as standard deviations, and the large standard deviations are due to the relative low measured currents. Note that each potential shows similar proton inventory behavior.

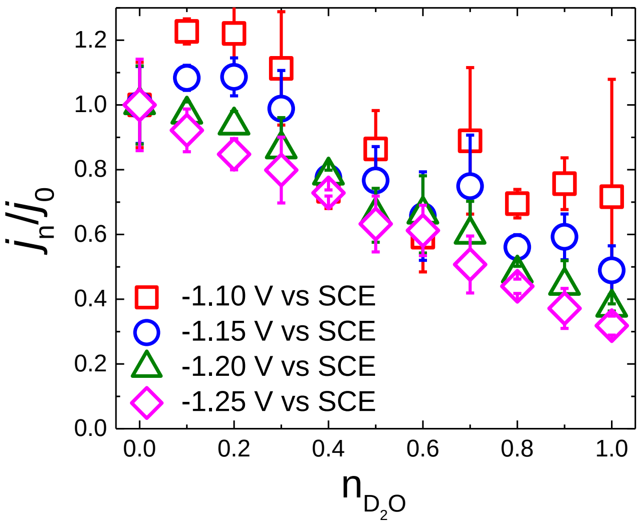

Supplementary Figure 34. Proton inventory measurements of CO_2 reduction by CoPc(py) at different potentials based on chronoamperometric (CA) steps. Note: Data at −1.00 V and −1.05 V vs. SCE were not included because minimal catalytic current is observed at those potentials. All reported values are averages from 3 or more independent measurements, all errors are given as standard deviations, and the large standard deviations are due to the relative low measured currents. Note that each potential shows similar proton inventory behavior.

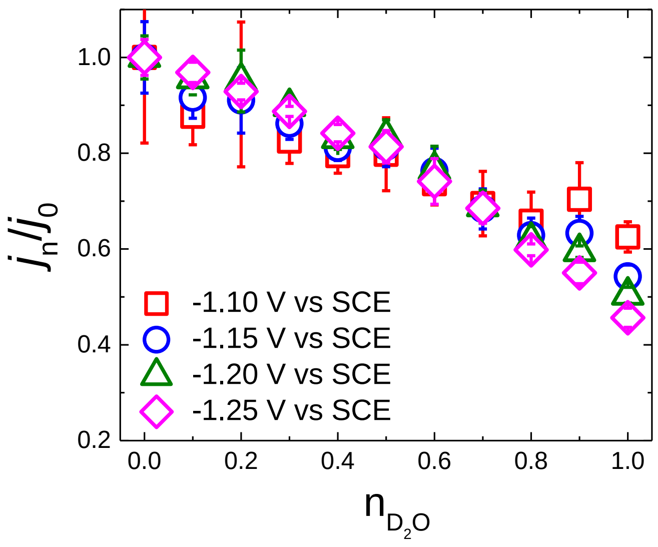

Supplementary Figure 35. Proton inventory measurements of CO_2 reduction by CoPc-0.5 %P4VP at different potentials based on chronoamperometric (CA) steps. Note: Data at −1.00 V and −1.05 V vs. SCE were not included because minimal catalytic current is observed at those potentials. All reported values are averages from 3 or more independent measurements, all errors are given as standard deviations, and the large standard deviations are due to the relative low measured currents. Note that each potential shows similar proton inventory behavior.

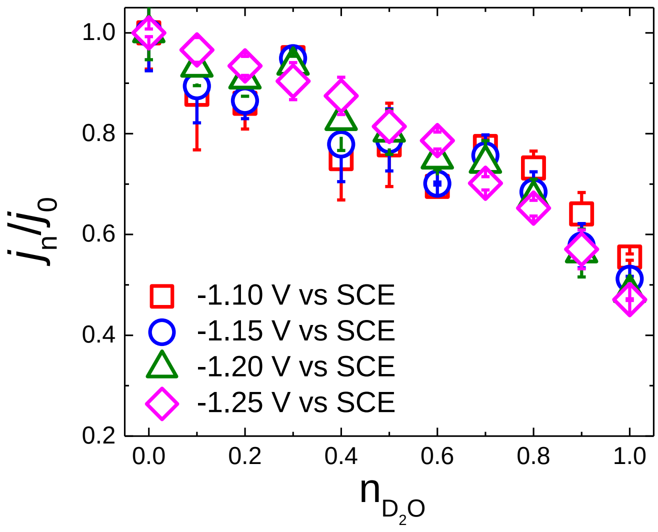

Supplementary Figure 36. Proton inventory measurements of CO_2 reduction by CoPc-1% P4VP at different potentials based on chronoamperometric (CA) steps. Note: Data at −1.00 V and −1.05 V vs. SCE were not included because minimal catalytic current is observed at those potentials. All reported values are averages from 3 or more independent measurements, all errors are given as standard deviations, and the large standard deviations are due to the relative low measured currents. Note that each potential shows similar proton inventory behavior.

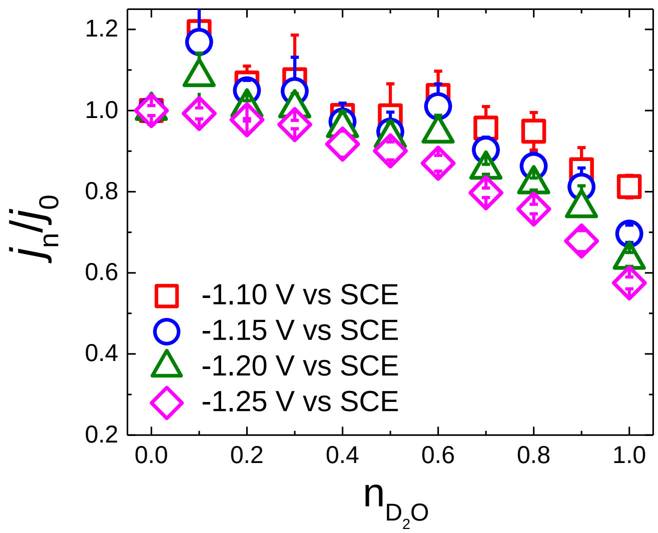

Supplementary Figure 37. Proton inventory measurements of CO_2 reduction by CoPc-3% P4VP at different potentials based on chronoamperometric (CA) steps. Note: Data at −1.00 V and −1.05 V vs. SCE were not included because minimal catalytic current is observed at those potentials. All reported values are averages from 3 or more independent measurements, all errors are given as standard deviations, and the large standard deviations are due to the relative low measured currents. Note that each potential shows similar proton inventory behavior.

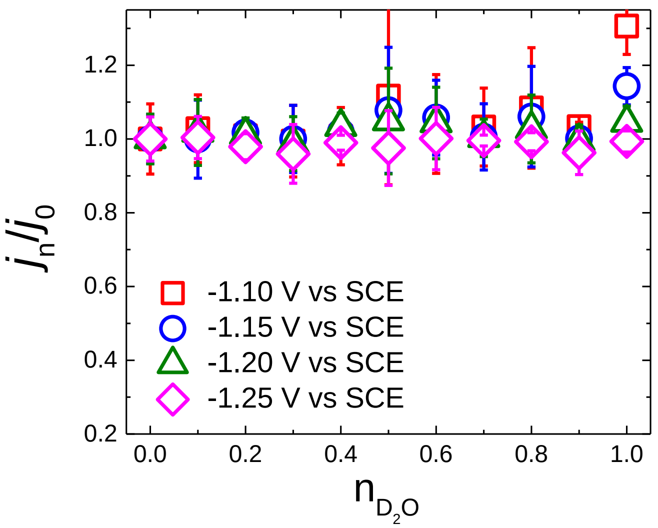

Supplementary Figure 38. Proton inventory measurements of CO_2 reduction by CoPc-P2VP at different potentials based on chronoamperometric (CA) steps. Note: Data at −1.00 V and −1.05 V vs. SCE were not included because minimal catalytic current is observed at those potentials. All reported values are averages from 3 or more independent measurements, all errors are given as standard deviations, and the large standard deviations are due to the relative low measured currents. Note that each potential shows similar proton inventory behavior.

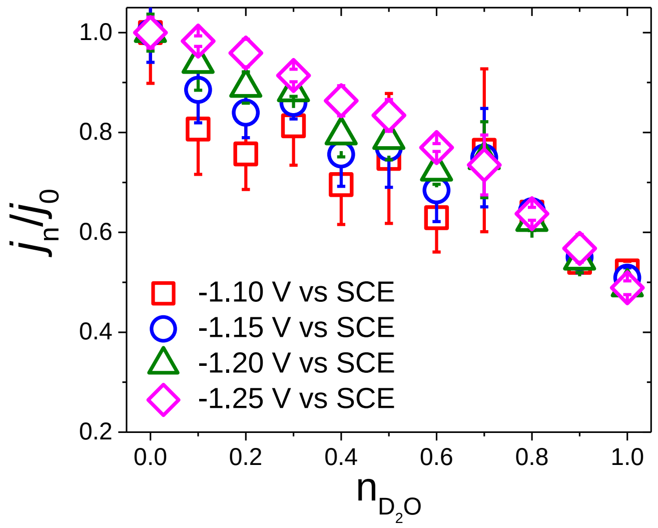

Supplementary Figure 39. Proton inventory measurements of CO_2 reduction by CoPc(py)-P2VP at different potentials based on chronoamperometric (CA) steps. Note: Data at −1.00 V and −1.05 V vs. SCE were not included because minimal catalytic current is observed at those potentials. All reported values are averages from 3 or more independent measurements, all errors are given as standard deviations, and the large standard deviations are due to the relative low measured currents. Note that each potential shows similar proton inventory behavior.

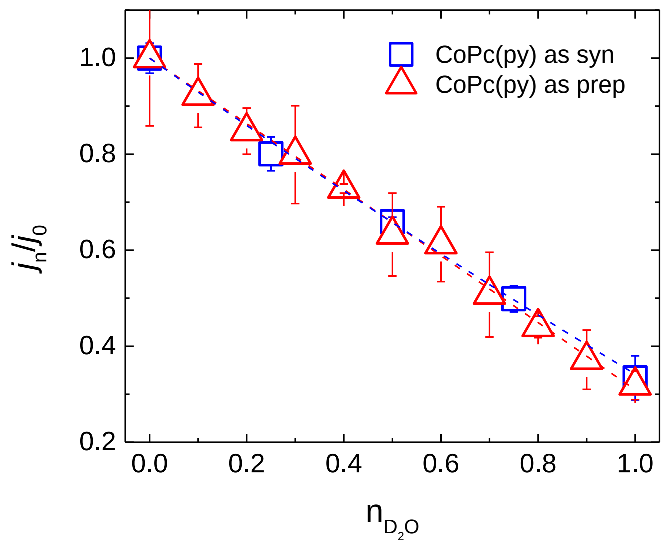

Supplementary Figure 40. Proton Inventory measurement of CO_2 reduction at -1.25 V vs SCE by CoPc(py) films prepared two different ways: by method 1 (CoPc and pyridine mixed in DMF and then dropcast) (red triangles) and method 2 (CoPc(py) synthesized independently and then dropcast from DMF) (blue squares). The red and blue dashed lines are fit to the data using equation (2). The resulting values for ϕ and Z are shown in Supplementary Table 8. Note that KIE $=j_H/j_D$. All reported values are averages from 3 or more independent measurements, and all errors are given as standard deviations.

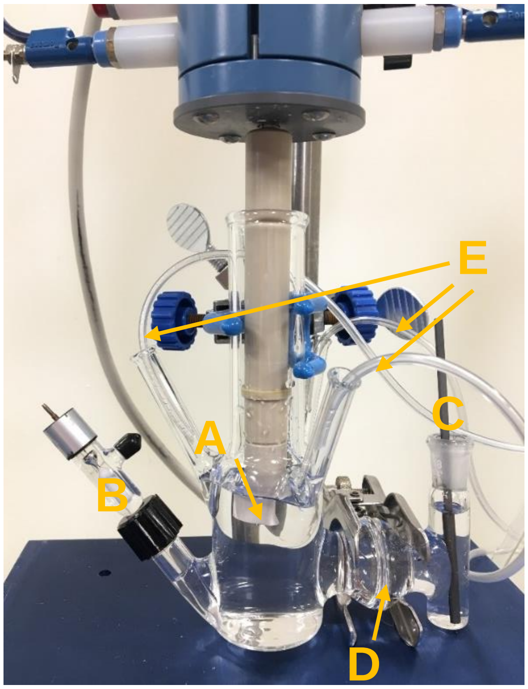

Supplementary Figure 41. Photograph of a custom two-compartment glass cell: (A) Modified working electrode held in a RDE assembly attached to an MSR rotator, (B) Sealed SCE reference electrode, (C) Carbon rod auxiliary electrode, (D) Nafion-117 membrane, (E) Tygon tubings for delivering CO_2 gas to blanket headspace of the cell. In general, gas-tight seals were made either by O-ring compression.

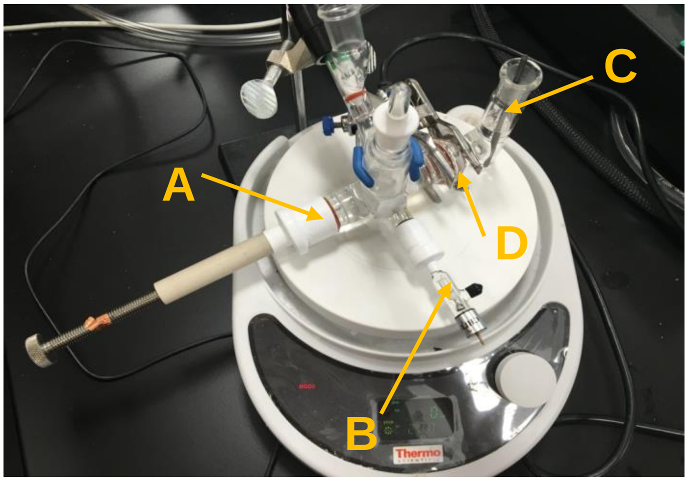

Supplementary Figure 42. Photograph of custom, gas-tight two-chamber U-cell: (A) Modified working electrode held in a RDE internal hardware kit and mounted into a custom PEEK sleeve, (B) Sealed SCE reference electrode, (C) Carbon rod auxiliary electrode, (D) Nafion-117 membrane. In general, gas-tight seals were made either by O-ring compression or with ground-glass joints.

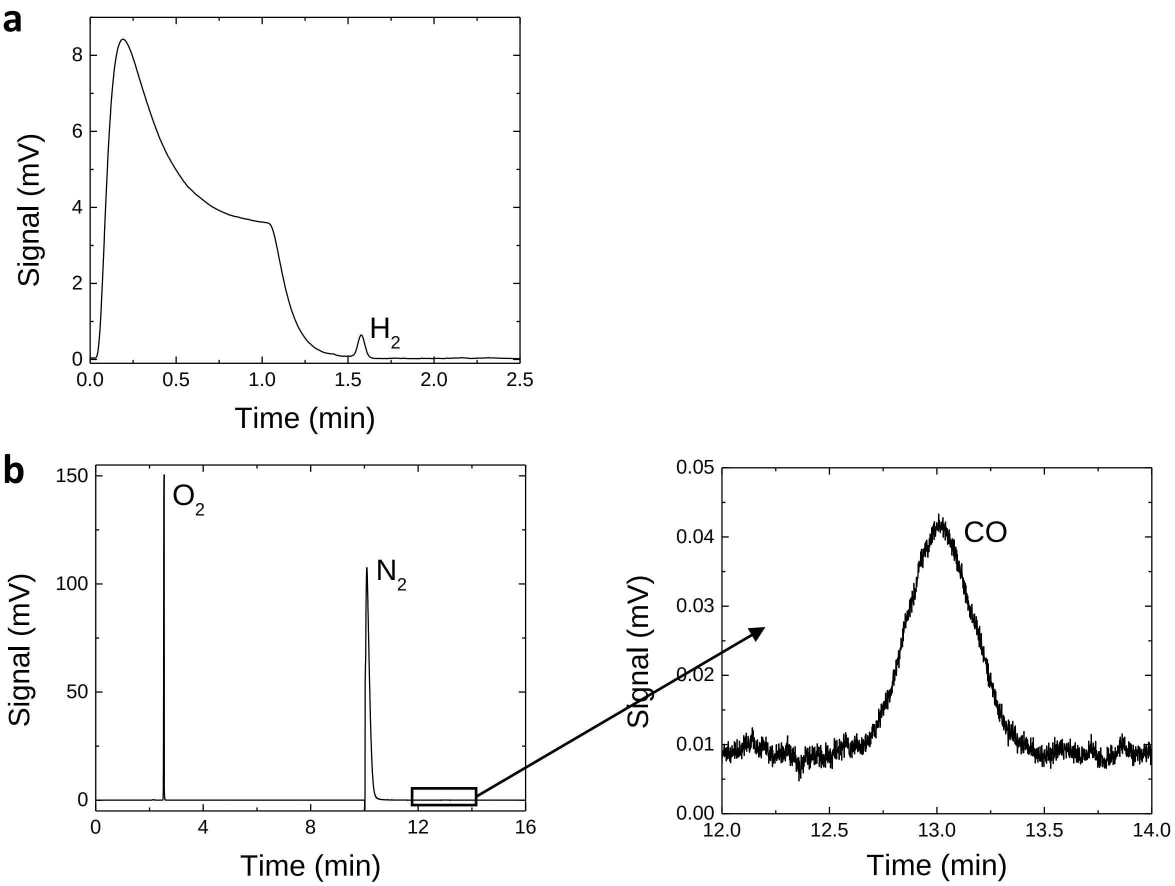

Supplementary Figure 43. Representative chromatograph of a calibration mixture containing 0.05 % H_2, 0.05 % CO, 99.9 % N_2. Note: the signal (retention time from 0 to 1 min) before H_2 peak in **a** is due to GC valve switching.

a, Front TCD.

b, Back TCD.

Supplementary Tables

Supplementary Table 1. Co loading of pre- and post-chronoamperometric (CA) measurement

Catalyst	Pre-CA measurement		Post-CA measurement	
	**Co concentration (ppb)**	**Co loading (×10^{-9} mol cm^{-2})**	**Co concentration (ppb)**	**Co loading (×10^{-9} mol cm^{-2})**
CoPc	2.74 ± 0.26	2.04 ± 0.19	2.89 ± 0.17	2.15 ± 0.13
CoPc(py)	2.44 ± 0.27	1.81 ± 0.20	2.60 ± 0.25	1.93 ± 0.18
CoPc-P2VP	2.40 ± 0.06	1.78 ± 0.05	2.85 ± 0.22	2.11 ± 0.17
CoPc-0.1 % P4VP	2.88 ± 0.47	2.14 ± 0.35	2.85 ± 0.34	2.12 ± 0.26
CoPc-0.5 % P4VP	2.74 ± 0.22	2.03 ± 0.16	2.71 ± 0.57	2.01 ± 0.42
CoPc-1 % P4VP	2.84 ± 0.28	2.11 ± 0.21	3.02 ± 1.28	2.25 ± 0.95
CoPc-2 % P4VP	2.88 ± 0.13	2.14 ± 0.09	2.80 ± 0.28	2.08 ± 0.21
CoPc-3 % P4VP	2.75 ± 0.35	2.04 ± 0.26	2.98 ± 0.15	2.21 ± 0.11
CoPc(py)-P2VP	2.80 ± 0.50	2.08 ± 0.37	2.82 ± 0.19	2.10 ± 0.14
CoPc-PS	2.79 ± 0.17	2.07 ± 0.13	2.82 ± 0.09	2.09 ± 0.07
CoPc(py)-PS	2.85 ± 0.11	2.12 ± 0.08	2.80 ± 0.41	2.08 ± 0.31

The catalyst films were dissolved from the electrode surface in 5 mL of 1 M nitric acid solution and then ICP-MS measurements were conducted. The catalyst loading was calculated based on the dissolution solution volume and the concentration. Because ICP-MS measurements require dissolution of the catalyst film from the electrode surface, it is not possible to confirm the loading of the same electrode Pre-CA and Post-CA. Instead, Co loadings were measured on identically-prepared electrodes pre-CA and post-CA (see Supplementary Methods). The errors are given as standard deviations on three identically prepared electrodes.

Supplementary Table 2. Results obtained from 2-h controlled potential electrolyses (CPE) experiments at -1.25 V vs SCE for CoPc modified electrodes in pH 5 protic phosphate solution and pD 5 deuterated phosphate solution for CO_2 reduction.

Catalyst	Solvent	Charge (C)	ε_{CO} (%)	TON_{CO} (2h)	TOF_{CO} (s^{-1})	ε_{H2} (%)	ε_{total} (%)
CoPc	H_2O	0.62 ± 0.07	60 ± 3	7.8 ± 1.2 ×10^3	1.08 ± 0.17	33 ± 6	93 ± 5
	D_2O	0.61 ± 0.05	58 ± 3	7.5 ± 0.7 ×10^3	1.04 ± 0.10	36 ± 1	95 ± 4
CoPc(py)	H_2O	1.09 ± 0.02	78 ± 4	1.8 ± 0.1 ×10^4	2.44 ± 0.13	11 ± 2	89 ± 2
	D_2O	0.32 ± 0.06	76 ± 2	5.1 ± 0.8 ×10^3	0.71 ± 0.12	11 ± 1	87 ± 3
CoPc-0.1 %P4VP	H_2O	1.07 ± 0.17	89 ± 2	2.0 ± 0.3 ×10^4	2.72 ± 0.40	7 ± 2	96 ± 1
	D_2O	0.47 ± 0.07	90 ± 2	8.8 ± 1.6 ×10^3	1.23 ± 0.22	8 ± 1	98 ± 2
CoPc-0.5 %P4VP	H_2O	1.31 ± 0.10	92 ± 3	2.5 ± 0.1 ×10^4	3.45 ± 0.17	8 ± 4	99 ± 4
	D_2O	0.62 ± 0.04	90 ± 5	1.2 ± 0.1 ×10^4	1.62 ± 0.15	7 ± 1	98 ± 5
CoPc-1 %P4VP	H_2O	1.57 ± 0.09	92 ± 1	3.0 ± 0.2 ×10^4	4.16 ± 0.21	8 ± 2	100 ± 2
	D_2O	0.78 ± 0.03	96 ± 5	1.6 ± 0.1 ×10^4	2.16 ± 0.14	5 ± 1	101 ± 4
CoPc-2 %P4VP	H_2O	1.97 ± 0.25	94 ± 3	3.8 ± 0.5 ×10^4	5.31 ± 0.65	5 ± 2	99 ± 3
	D_2O	1.10 ± 0.13	89 ± 3	2.0 ± 0.2 ×10^4	2.81 ± 0.30	9 ± 1	98 ± 4
CoPc-3 %P4VP	H_2O	2.28 ± 0.10	93 ± 2	4.4 ± 0.1 ×10^4	6.09 ± 0.16	6 ± 1	99 ± 2
	D_2O	1.27 ± 0.11	91 ± 2	2.4 ± 0.2 ×10^4	3.31 ± 0.28	10 ± 2	100 ± 2
CoPc-P2VP[a]	H_2O	0.40 ± 0.08	82 ± 2	6.9 ± 1.4 ×10^3	0.95 ± 0.20	14 ± 5	97 ± 3
	D_2O	0.41 ± 0.03	82 ± 1	6.9 ± 0.6 ×10^3	0.96 ± 0.08	12 ± 4	94 ± 4
CoPc(py)-P2VP[a]	H_2O	1.56 ± 0.04	91 ± 1	2.9 ± 0.1 ×10^4	4.08 ± 0.13	6 ± 1	97 ± 1
	D_2O	0.81 ± 0.11	90 ± 3	1.5 ± 0.2 ×10^4	2.08 ± 0.33	3 ± 1	93 ± 2
CoPc-PS[a]	H_2O	0.14 ± 0.06	86 ± 2	2.5 ± 1.4 ×10^3	0.35 ± 0.19	15 ± 2	100 ± 1
	D_2O	0.18 ± 0.06	81 ± 2	3.0 ± 1.0 ×10^3	0.41 ± 0.14	18 ± 4	100 ± 6
CoPc(py)-PS[a]	H_2O	0.20 ± 0.03	86 ± 4	3.6 ± 0.5 ×10^3	0.50 ± 0.07	7 ± 2	93 ± 2
	D_2O	0.07 ± 0.01	87 ± 3	1.2 ± 0.2 ×10^3	0.16 ± 0.03	14 ± 1	95 ± 3

All reported values are averages from 3 independent measurements, and all errors are given as standard deviations. [a] Polymer-catalyst composite films were drop-cast from deposition solutions containing 1 % w/v polymer.

Supplementary Table 3. Results obtained from 4-h controlled potential electrolyses (CPE) experiments at -1.25 V vs SCE for CoPc-P4VP catalyst in pH 5 phosphate solution under CO_2 atmosphere.

Catalyst	Charge(C)	ε_{CO} (%)	TON_{CO} (2h)	TOF_{CO} (s^{-1})	ε_{H2} (%)	ε_{total} (%)
CoPc-P4VP	3.77 ± 0.30	91 ± 3	7.1 ± 0.5 $\times 10^4$	4.93 ± 0.34	10 ± 1	101 ± 4
CoPc-P4VP[a]	3.84	91	7.21 $\times 10^4$	5.01	9	99

All reported values are averages from 3 or more independent measurements, and all errors are given as standard deviations. [a]Continue another 4-h CPE with the same catalyst after re-saturate the same electrolyte with CO_2.

Supplementary Table 4. Results of kinetic isotope effect (KIE) studies for CoPc, CoPc(py), CoPc-P2VP, CoPc-P4VP, and CoPc-PS catalyst for hydrogen evolution reaction (HER).

**Catalyst**	**j_H (mA·cm^{-2})**	**j_D (mA·cm^{-2})**	**KIE**
CoPc	-1.856 ± 0.158	-1.349 ± 0.241	1.38 ± 0.27
CoP(py)	-1.495 ± 0.155	-1.058 ± 0.228	1.41 ± 0.34
CoPc-P2VP	-1.397 ± 0.060	-1.066 ± 0.078	1.31 ± 0.11
CoPc-P4VP	-2.018 ± 0.195	-1.458 ± 0.021	1.38 ± 0.14
CoPc-PS	-0.277 ± 0.032	-0.209 ± 0.018	1.33 ± 0.19

All measurements conducted using rotating disk chronoamperometric (CA) measurements under N_2 in pH/pD = 4.7 (the same pH/pD of the electrolyte in CO_2 reduction measurements after saturated by CO_2) phosphate solutions at -1.25 V vs. SCE. All reported values are averages from 3 or more independent measurements, and all errors are given as standard deviations. Note that a weak KIE for HER has been observed for all the catalytic systems studied in this study, which suggests that the rate-determining step for HER may be the proton recombination step on the 2 H^+ protonated CoPc intermediate.[4]

Supplementary Table 5. Results of kinetic isotope effect (KIE) studies for CoPc and CoPc-P4VP at different CoPc loadings.

**CoPc loading (mol cm^{-2})**	**Catalyst**	**j_H (mA·cm^{-2})**	**j_D (mA·cm^{-2})**	**$\varepsilon_{CO,H}$ (%)**	**$\varepsilon_{CO,D}$ (%)**	**KIE**
2.19×10^{-11} [a]	CoPc	-0.31 ± 0.01	-0.32 ± 0.02	70 ± 1	66 ± 9	1.0 ± 0.1
	CoPc-P4VP	-1.56 ± 0.22	-0.88 ± 0.11	92 ± 2	91 ± 3	1.8 ± 0.3
2.19×10^{-9} [b]	CoPc	-0.87 ± 0.07	-0.88 ± 0.12	60 ± 3	58 ± 3	1.0 ± 0.2
	CoPc-P4VP	-2.90 ± 0.02	-1.37 ± 0.01	92 ± 1	96 ± 5	2.1 ± 0.1

Current density (*j*) measurements were conducted through rotating disk chronoamperometric (CA) step measurements, and Faradaic efficiency (ε) measurements were conducted through 2-h controlled potential electrolyses (CPE). All measurements were conducted at -1.25 V vs. SCE in pH/pD 5 phosphate solution under CO_2 atmosphere. All reported values are averages from 3 or more independent measurements, and all errors are given as standard deviations. [a] The lowest loading used in the loading dependence study. [b] Normal loading used in this work for comparison.

Supplementary Table 6. Results obtained from 2-h controlled potential electrolyses (CPE) experiments at -1.25 V vs SCE for bare EPG working electrodes in pH 5 phosphate solution under CO_2 atmosphere with 0.05 mM added pyridine (py). Results for CPE experiments with CoPc without added py and CoPc(py) are included for comparison.

Catalyst	Charge(C)	ε_{CO} (%)	TON_{CO} (2h)	TOF_{CO} (s^{-1})	ε_{H2} (%)	ε_{total} (%)
0.05 mM py	0.27 ± 0.03	4 ± 2	$5.2 \pm 2.8 \times 10^{-2}$	$7.2 \pm 3.8 \times 10^{-2}$	62 ± 11	66 ± 12
CoPc	0.62 ± 0.07	60 ± 3	$7.8 \pm 1.2 \times 10^{3}$	1.08 ± 0.17	33 ± 6	93 ± 5
CoPc(py)	1.09 ± 0.02	78 ± 4	$1.8 \pm 0.1 \times 10^{4}$	2.44 ± 0.13	11 ± 2	89 ± 2

Note that there's no liquid products detected in any of the above catalytic systems. All reported values are averages from 3 or more independent measurements, and all errors are given as standard deviations.

Supplementary Table 7. Elemental Analysis results for synthesized CoPc(py) complex

Element	Theory (%)	Found (%)
C	68.30	67.98
H	3.25	3.26
N	19.38	19.21

Supplementary Table 8. Results of kinetic isotope effect (KIE) studies and proton inventory measurements for CoPc(py) as synthesized, results of proton inventory measurements for CoPc(py) as prepared is included for comparison.

**Catalyst**	**j_H (mA·cm^{-2})**	**j_D (mA·cm^{-2})**	**KIE**	**Proton Inventory Parameters**	
				**ϕ**	**Z**
CoPc(py) as synthesized	-2.02 ± 0.06	-0.67 ± 0.09	3.0 ± 0.4	0.37 ± 0.04	0.92 ± 0.06
CoPc(py) as prepared	-1.92 ± 0.27	-0.61 ± 0.06	3.1 ± 0.5	0.30 ± 0.01	1.02 ± 0.02

All measurements conducted using rotating disk chronoamperometric (CA) measurements at -1.25 V vs. SCE in pH/pD 5 phosphate solution under CO_2 atmosphere. All reported values are averages from 3 or more independent measurements, and all errors are given as standard deviations.

Supplementary Methods

Materials and Chemicals

All purchased chemicals were used as received unless otherwise specified. Cobalt phthalocyanine (CoPc, 97%), poly-4-vinylpyridine (P4VP, average Mw ~ 160,000), poly-2-vinylpyridine (P2VP, average Mw ~ 159,000), N,N-Dimethylformamide (DMF, ACS grade), pyridine (ACS grade, ≥ 99%), Dimethyl sulfoxide (DMSO, ACS grade, ≥ 99.9 %), 2,2,2-trifluoroethanol (TFE, ReagentPlus grade, ≥ 99.0%), ferrocenecarboxylic acid (97%), sodium phosphate monobasic (BioXtra, > 99.0%), sodium deuteroxide solution (NaOD, 40 wt.% in D_2O, 99.5% D), phosphoric acid-D3 solution (85 wt.% in D_2O), and Nafion-117 cation exchange membrane (Nafion) were purchased from Sigma Aldrich. Deuterium oxide was purchased from both Sigma Aldrich (D, 99.9%, Mw = 20.03) and Cambridge Isotope Labs, Inc. (D, 99.9%, Mw = 20.03). Sulfuric acid (TraceMetal grade) and nitric acid (TraceMetal grade, 67-70 %) were purchased from Fisher Scientific. Tetrabutylammonium hexaflurophosphate (nBu_4NPF_6, > 98.0%) was purchased from TCI America and recrystallized from Methonal/H_2O (v/v = 8/1). Cobalt ICP standard (1000 ppm Co in 3 % HNO_3) was purchased from Ricca Chemical Company. Edge-plane graphite disk electrodes (5 mm diameter, effective electrode area: 0.114 cm^2) were purchased from Pine Research Instrumentation. Pt wire (99.99 %, 0.02" diameter) was purchased from Surepure Chemetals L.L.C. Compressed CO_2 gas (99.8 %) was purchased from Cryogenic Gases. All water used in this study was ultrapure water (18.2 MΩ·cm resistivity) purified with a Thermo Scientific GenPure UV-TOC/UF xCAD-plus water purification system. Plain glass microscope slides were purchased from Fisher Scientific.

Preparation of Deposition Solutions

CoPc. The CoPc/DMF deposition solution was prepared by dissolving 3 mg of CoPc in 100 mL DMF solvent. The resulting solution was sonicated for 30 min to fully disperse the CoPc. The concentration of CoPc in the resulting solution is 0.05 mM.

CoPc(py). The CoPc(py)/DMF deposition solution was prepared two ways:

1. **Method 1:** By dissolving 3 mg of CoPc in a mixture of 95 mL DMF and 5 mL of pyridine (19:1 DMF/pyridine). The resulting solution was sonicated for 30 min to fully disperse the CoPc. The concentration of CoPc(py) in the resulting solution is 0.05 mM.
2. **Method 2:** By first synthesizing CoPc(py) based on previously-reported procedures.[6] Synthesis of 5-coordinate CoPc(py) was confirmed by Elemental Analysis conducted by Midwest Microlab, Inc. (see Supplementary Table 7). Films were prepared by dissolving 3.3 mg CoPc(py) as synthesized in 100 mL DMF, then the solution was sonicated for 30 min, the concentration of CoPc(py) as synthesized in the resulting solution is 0.05 mM.

Drop-cast films prepared by both methods showed analogous KIE and proton inventory results (see Supplementary Figure 40 and Supplementary Table 8), suggesting the prepared films are identical. In addition, liquid-phase UV-Vis measurements of CoPc(py) deposition solutions prepared by both methods show an analogous red-shift in the UV-Vis spectra suggesting both are 5-coordinate species (see Supplementary Figure 29)

CoPc-P4VP. The CoPc-P4VP (CoPc-1% P4VP) deposition solution was prepared by dissolving 0.01 g of P4VP in 1 mL of CoPc/DMF solution. The resulting solution was sonicated for 20 mins to fully dissolve the P4VP and disperse the CoPc. The CoPc-0.1% P4VP and CoPc-0.5% P4VP solutions were prepared by diluting the CoPc-1% P4VP solution by a factor of 10 and a factor of 2, respectively. CoPc-2 % P4VP and CoPc-3 % P4VP deposition solutions were prepared by dissolving 0.02 g P4VP and 0.03 g P4VP in 1 mL CoPc/DMF solution, respectively. The resulting solution was sonicated for 20 mins to fully dissolve the P4VP and disperse the CoPc.

CoPc-P2VP/DMF. The CoPc-P2VP deposition solution was prepared by dissolving 0.01 g P2VP in 1 mL of CoPc/DMF solution. The resulting solution was sonicated for 20 mins to fully dissolve the P2VP and disperse the CoPc.

CoPc(py)-P2VP. The CoPc(py)-P2VP deposition solution was prepared by dissolving 0.01 g P2VP in 1 mL of CoPc(py)/DMF solution. The resulting solution was sonicated for 20 mins to fully dissolve the P2VP and disperse the CoPc.

CoPc-PS. The CoPc-PS deposition solution was prepared by dissolving 0.01 g PS in 1 mL CoPc/DMF solution. The resulting solution was sonicated for 20 mins to fully dissolve the PS and disperse the CoPc.

CoPc(py)-PS. The CoPc(py)-PS deposition solution was prepared by dissolving 0.01 g PS in 1 mL CoPc(py)/DMF solution. The resulting solution was sonicated for 20 mins to fully dissolve the PS and disperse the CoPc.

Scan Rate Dependence Studies

Scan rate dependence studies were conducted in pH 5 phosphate solution using the same cell described in the main text (see Methods in the main text). The electrolyte solution was degassed with N_2 for ~ 30 min prior to the measurement, and the headspace was blanked with N_2 during the measurement. Rotating disk cyclic voltammetry measurement was conducted for all the catalyst systems investigated in this work at 1600 rpm with the potential range from 0.8 V to -0.8 V vs. SCE. The scan rate was varied from 0.2 V s^{-1} to 6.4 V s^{-1} (see Supplementary Figure 1−Supplementary Figure 14).

ICP-MS Measurement

To measure the Co loading on the film-modified electrodes, as-prepared electrodes and identically-prepared electrodes after the rotating disk chronoamperometric (CA) step measurements were submerged in individual scintillation vials containing 4.5 mL of 1 M nitric acid aqueous solution. The vials containing the electrodes and nitric acid solutions were placed onto a vortex mixer (Fisher Scientific) at 500 rpm for 3 h. The electrodes were then removed from the vials, and the resulting solutions were diluted with 0.5 mL ultrapure water to a final volume of 5 mL. The diluted samples were then analyzed for Co concentration using a Perkin-Elmer Nexion 2000 ICP-MS instrument. The instrument was calibrated using cobalt calibration standards at concentrations of 1 ppb, 3ppb, and 5 ppb which were prepared from dilution of a 1000 ppm standard (Ricca Chemical Company). The results of ICP-MS measurement are summarized in Supplementary Table 1.

CoPc Loading Dependence Study

CoPc catalyst deposition solution was prepared by dispersing ~ 3 mg of CoPc in 1 mL of DMF with 30 min of sonication. This solution was then serially diluted in DMF to obtain catalyst deposition solutions containing CoPc concentrations ranging from 3×10^{-4} mg mL^{-1} to 3 mg mL^{-1}. CoPc-1% P4VP catalyst deposition solutions was prepared by dissolving 0.01 g of P4VP in each of 1 mL of CoPc deposition solutions with 20 min of sonication. The modified EPG working electrode was then prepared by drop-casting 5 µL of the prepared deposition solution onto EPG. The electrode was then oven-dried in air at 70 ° for 15 min to allow solvent to evaporate. The resulting loading of CoPc on the electrode surface ranges from 2.19×10^{-11} to 2.19×10^{-7} mol cm^{-2}.

Sample Preparation for UV-vis Spectroscopy

Solid-state and liquid samples were analyzed using Varian Cary 100 Bio UV-Visble Spectrophotometer. Solid-state CoPc-PS, CoPc-P4VP, CoPc-P2VP, CoPc(py)-PS and CoPc(py)-P2VP films were prepared by drop-casting total volume of 1 mL of the corresponding deposition solution (see Preparation of Deposition Solutions in the Supplementary Methods) on a 0.9 cm by 3 cm glass slide cut from the plain microscope slide, the DMF solvent was evaporated at 70 °C in an oven. The glass slide was then put into a glass cuvette in the UV-vis spectrometer for measurement. 0.01 mM CoPc/DMF solution and 0.01 mM CoPc(py) solutions are prepared by diluting the corresponding deposition solution by 5 times (see Preparation of Deposition Solutions in the Supplementary Methods).

Cyclic Voltammetry

The working electrode was a 0.071 cm^2 glassy carbon disk electrode (CH instruments), and the counter electrode was Pt wire (99.99 %, Surepure Chemetals L.L.C.). The reference electrode was a Ag/$AgNO_3$ (1.0 mM)/DMSO nonaqueous reference electrode, separated from the solution by a Vycor frit (Bioanalytical Systems, Inc.) and externally referenced to ferrocene. The scan rate was 50 mV s^{-1}. Cyclic voltammograms

were automatically corrected for IR drop at 85% through positive feedback using the Bio-Logic EC Lab software. DMSO solutions containing 0.1 M nBu_4NPF_6 were thoroughly degassed with N_2 or CO_2 for 20 min prior to measurements and a N_2 or CO_2 atmosphere was kept over the solutions during experiments.

Explanation of Equation (2) in Main Text:

Equation (2) in the main text , which was used to fit our proton inventory data, was derived from the Kresge-Gross-Butler equation (Supplementary Equation (1)),[7,8] which represents the isotope effect arises from a combination of pronounced isotope effect at a few sites (i.e., these sites have ∅ values that are quite different than unity), and from a *Z*-effect (i.e. these sites have ∅ values that are very close to unity individually but has an aggregate isotope effect as a whole):[7]

$$k_n = k_0 \left[\frac{\prod_{i=1}^{x}(1-n+n\emptyset_{Ti})}{\prod_{i=1}^{x}(1-n+n\emptyset_{Ri})}\right] Z^n \qquad (1)$$

where k_0 is the kinetic rate constant in protic solution, k_n is the kinetic rate constant in a solution containing a mole fraction of D_2O of *n*, x is the number of hydrogenic sites in the reactant or transition state, $\emptyset_{Ti}$ and $\emptyset_{Ri}$ are the isotopic fractionation factor for hydrogenic site in the transition- and reactant-state, respectively. And *Z* is given by Supplementary Equation (2):[9]

$$Z = \exp\left[-\gamma\left(1-\emptyset_{T,Z}\right) + \mu\left(1-\emptyset_{R,Z}\right)\right] \qquad (2)$$

Z^n reflects the *Z*-effect, or the solvent isotope effect that arises from small contributions at a large number of identical hydrogenic sites. These large number of hydrogenic sites can occur either only in the transition-state ($\emptyset_{R,Z} = 1, \emptyset_{T,Z} \neq 1$), the reactant-state ($\emptyset_{T,Z} = 1, \emptyset_{,R,Z} \neq 1$), or from a combination in the reactant- and transition-states).[7] μ and γ are the number of hydrogenic sites in the reactant- and transition-state from *Z*-sites, respectively.[9] Note that when $Z = 1$ then $\emptyset_{R,Z} = \emptyset_{T,Z} = 1$ and there are no *Z*-sites that contribute to the isotope effect. When $Z > 1$, then the Z-sites contribute an inverse isotope effect, and when $Z < 1$ then *Z*-sites contribute a normal isotope effect.[9]

In our case, the pronounced isotope effect occurs at a single hydrogenic site involved in step (iii) in Fig. 3a in the main text, thus, Supplementary Equation (1) reduces to:

$$k_n = k_0 \left(\frac{1-n+n\emptyset_T}{1-n+n\emptyset_R}\right) Z^n \qquad (3)$$

If we assume that the reactant-state fractionation factor $\emptyset_R$ for the hydrogen attached to the oxygen of CO_2 molecule is unity,[10] then Supplementary Equation (3) becomes:

$$k_n = k_0\,(1-n+n\emptyset)Z^n \qquad (4)$$

Where ∅ is the isotopic fractionation factor for hydrogenic site involved in step (iii) in Fig. 3a (in main text) in the transition-state. Combining Supplementary Equation (4) and equation ($\mathrm{KIE} = \frac{k_\mathrm{H}}{k_\mathrm{D}} = \frac{j_\mathrm{H}}{j_\mathrm{D}}$, main text) produce equation:

$$j_n = j_0\,(1-n+n\emptyset)Z^n \qquad (5)$$

Turnover Frequency for CO (TOF_{co}) Calculations for Rotating Disk Chronoamperometric (CA) Steps Measurements

TOF_{CO} was calculated based on the total amount of CO generated divided by the total amount of the catalyst on the electrode and the time of the electrolysis.

$$\mathrm{TOF_{CO}} = \frac{\frac{\mathrm{n_{CO}}}{\mathrm{n_{cat}}}}{\mathrm{t}} \qquad (6)$$

$\mathrm{n_{CO}}$ is the total number of moles of CO produced, $\mathrm{n_{cat}}$ is the number of moles of the catalyst on the electrode surface, and t is the electrolysis time in seconds.
$\mathrm{n_{CO}}$ is calculated based on the amount of electrons used specifically for CO generation divided by a factor of 2F (it is a two-electron reduction reaction from CO_2 to CO):

$$\mathrm{n_{CO}} = \frac{\mathrm{Q} \times \varepsilon_{\mathrm{CO}}}{2\mathrm{F}} \qquad (7)$$

Q is the charge passed in Coulombs (C); $\varepsilon_{\mathrm{CO}}$ is the Faradaic Efficiency of CO in percentage (%) obtained in controlled potential electrolyses (CPE) experiments; F is Faraday constant ($\mathrm{C\ mol^{-1}}$).
$\mathrm{n_{cat}}$ is calculated based on the following equation:

$$\mathrm{n_{cat}} = [\mathrm{cat}] \times \mathrm{V} \qquad (8)$$

[cat] is the concentration of the catalyst in the deposition solution ($\mathrm{mol\ L^{-1}}$); V is the volume of the deposition solution drop-cast on the electrode surface (L).
In a rotaing disk CA steps measurement, the observed current i (A) is given as:

$$i = \frac{\mathrm{Q}}{\mathrm{t}} \qquad (9)$$

Here, Q is the charge passed in each rotating disk CA step (C), and t is the time for each step in seconds.

Combining Supplementary Equation (6), (7) and (9) produces:

$$\mathrm{TOF_{CO}} = \frac{i \times \varepsilon_{\mathrm{CO}}}{2\mathrm{F} \times \mathrm{n_{cat}}} = \frac{j \times \mathrm{A} \times \varepsilon_{\mathrm{CO}}}{2\mathrm{F} \times \mathrm{n_{cat}}} \qquad (10)$$

Here, j is the current density for each rotating disk CA step (A cm^{-2}), and A is the geomtric surface area of the EPG electrode (0.114 cm^2). We used the current density at -1.25 V vs.SCE for TOF_{CO} calculations. Note that this TOF calculation uses j and $\varepsilon_{\mathrm{CO}}$ results are from two different types of measurements (rotating disk CA steps and 2-h CPE experiments, respectively) at the same reduction potential which is an inherent limitation to this approach and may lead to unaccounted for inaccuracies in this calculation.

Supplementary References

1. Dinçer, H. A., Koca, A., Gül, A. & Koçak, M. B. Novel phthalocyanines bearing both quaternizable and bulky substituents. *Dyes and Pigments* **76**, 825-831 (2008).
2. Bedioui, F., De Boysson, E., Devynck, J. & Balkus, K. J. Electrochemical behaviour of zeolite-encapsulated cobalt phthalocyanine complex in DMSO and DMF solutions. *J. Electroanal. Chem. Interfacial Electrochem.* **315**, 313-318 (1991).
3. Kozub, B. R. & Compton, R. G. Voltammetric studies of the redox mediator, cobalt phthalocyanine, with regard to its claimed electrocatalytic properties. *Sensors and Actuators B: Chemical* **147**, 350-358 (2010).
4. Zhao, F., Zhang, J., Abe, T., Wöhrle, D. & Kaneko, M. Electrocatalytic proton reduction by phthalocyanine cobalt derivatives incorporated in poly(4-vinylpyridine-co-styrene) film. *J. Mol. Catal. A: Chem.* **145**, 245-256 (1999).
5. DeCoursey, T. E. & Cherny, V. V. Deuterium Isotope Effects on Permeation and Gating of Proton Channels in Rat Alveolar Epithelium. *The Journal of General Physiology* **109**, 415 (1997).
6. Cariati, F., Galizzioli, D., Morazzoni, F. & Busetto, C. New adducts of phthalocyaninatocobalt(II) with pyridine and 4-methylpyridine and their vibrational, magnetic, and electronic properties. Part I. Reactivity towards oxygen. *J. Chem. Soc., Dalton Trans.*, 556-561 (1975).
7. Kohen, A. & Limbach, H.-H. *Isotope Effects in Chemistry and Biology*. (Taylor & Francis-CRC Press, Boca Raton, 2006).
8. Fitzpatrick, P. F. Combining solvent isotope effects with substrate isotope effects in mechanistic studies of alcohol and amine oxidation by enzymes. *Biochim. Biophys. Acta, Proteins Proteomics* **1854**, 1746-1755 (2015).
9. Schowen, R. L. The use of solvent isotope effects in the pursuit of enzyme mechanisms. *J. Labelled Compd. Radiopharm.* **50**, 1052-1062 (2007).
10. Venkatasubban, K. & Schowen, R. L. The Proton Inventory Techniqu. *Crit. Rev. Biochem.* **17**, 1-44 (1984).
